# The genetic legacy of extreme exploitation in a polar vertebrate

**DOI:** 10.1038/s41598-020-61560-8

**Published:** 2020-03-20

**Authors:** Anneke J. Paijmans, Martin A. Stoffel, Marthán N. Bester, Alison C. Cleary, P. J. Nico De Bruyn, Jaume Forcada, Michael E. Goebel, Simon D. Goldsworthy, Christophe Guinet, Christian Lydersen, Kit M. Kovacs, Andrew Lowther, Joseph I. Hoffman

**Affiliations:** 1 Department of Animal Behaviour, Bielefeld University, 33501 Bielefeld, Germany; 2 Present Address: Institute of Evolutionary Biology, University of Edinburgh, Edinburgh, EH9 3FL United Kingdom; 3 Department of Zoology and Entomology, Mammal Research Institute, University of Pretoria, Private Bag X20, Hatfield, 0028 South Africa; 4 Norwegian Polar Institute, Fram Centre, 9296 Tromsø, Norway; 5 Department of Natural Sciences, University of Agder, 4630 Kristiansand, Norway; 6 British Antarctic Survey, High Cross, Madingley Road, Cambridge, CB3 OET UK; 7 Antarctic Ecosystem Research Division, Southwest Fisheries Science Center, National Marine Fisheries, National Oceanographic and Atmospheric Administration, 8901 La Jolla Shores Drive, La Jolla, CA 92037 USA; 8 Present Address: Institute of Marine Science, University of California Santa Cruz, 1156 High Street, Santa Cruz, CA 95064 USA; 9 South Australian Research and Development Institute, 2 Hamra Avenue, West Beach, South Australia 5024 Australia; 10 School of Biological Sciences, The University of Adelaide, Adelaide, South Australia 5005 Australia; 11 Centre d’Etudes Biologiques de Chizé (CEBC), CNRS and Université de La Rochelle - UMR 7372, 79360 Villiers en Bois, France

**Keywords:** Population genetics, Genetic variation, Molecular ecology, Conservation biology

## Abstract

Understanding the effects of human exploitation on the genetic composition of wild populations is important for predicting species persistence and adaptive potential. We therefore investigated the genetic legacy of large-scale commercial harvesting by reconstructing, on a global scale, the recent demographic history of the Antarctic fur seal (*Arctocephalus gazella*), a species that was hunted to the brink of extinction by 18^th^ and 19^th^ century sealers. Molecular genetic data from over 2,000 individuals sampled from all eight major breeding locations across the species’ circumpolar geographic distribution, show that at least four relict populations around Antarctica survived commercial hunting. Coalescent simulations suggest that all of these populations experienced severe bottlenecks down to effective population sizes of around 150–200. Nevertheless, comparably high levels of neutral genetic variability were retained as these declines are unlikely to have been strong enough to deplete allelic richness by more than around 15%. These findings suggest that even dramatic short-term declines need not necessarily result in major losses of diversity, and explain the apparent contradiction between the high genetic diversity of this species and its extreme exploitation history.

## Introduction

Anthropogenic exploitation is a major threat to global biodiversity^1,2^. For example, hunting has decimated many terrestrial species from the plains buffalo to the passenger pigeon^3,4^, while over-fishing has resulted in the collapse of many fish and marine mammal stocks^5,6^, reducing the capacity of the world᾿s oceans to provide food and ecosystem services^7^. This depletion of natural capital has been accelerating in pace as human populations continue to grow^2^ while animal populations across the planet are declining and being driven to extinction at an unprecedented rate^8^.

Theoretical and experimental studies suggest that severe declines in the effective population size (*N*_e_) increase extinction risk not only because small populations are more sensitive to demographic and environmental stochasticity^9,10^ but also because the erosion of genetic diversity that accompanies severe demographic reductions can reduce population viability and adaptive potential^11,12^. However, the genetic consequences of anthropogenic exploitation are in general poorly understood^13^ with debate continuing over the contribution of genetic factors to extinction^11^. For example, comparative studies have shown that threatened species often have lower genetic diversity than non-threatened taxa^12,14^. However, this pattern does not necessarily imply a causal link between genetic diversity and viability, as many threatened species appear to have small ancestral population sizes, implying that they may not have been very abundant in the first place^14^.

A compelling approach for investigating the genetic consequences of exploitation on natural populations is to use highly polymorphic genetic markers such as microsatellites to reconstruct the demographic histories of species that were heavily hunted in the past. In particular, recent severe demographic declines or bottlenecks^15,16^ can be inferred by comparing the observed genetic diversity of a contemporary sample with the diversity expected under alternative historical scenarios simulated based on the coalescent^17–19^. Applied in a comparative context, this approach has shown that the intensity of recent bottlenecks can be influenced by species-specific traits such as breeding ecology and mating system variation^20^. However, our understanding of within-species variation in demographic histories remains limited because most studies of individual species are conducted with suboptimal sample sizes of both individuals and loci, and test for the presence or absence of bottlenecks rather than quantifying their intensity^21^. In order to understand species-wide responses to anthropogenic exploitation, studies are needed that combine exhaustive geographical sampling with modern computational methods capable of deriving quantitative estimates of demographic parameters, such as approximate Bayesian computation (ABC)^22,23^.

**Figure 1:**
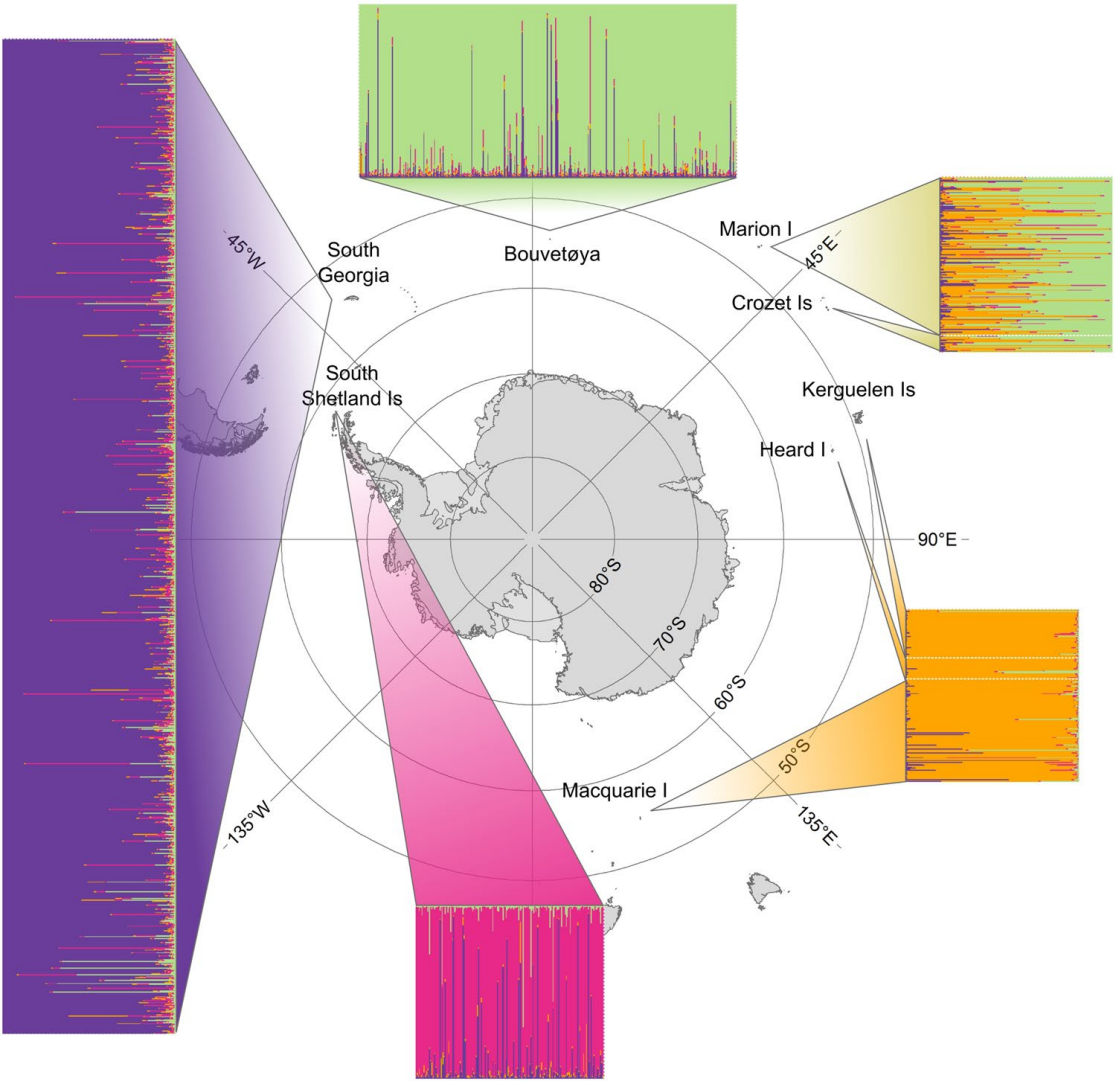
Global population structure of the Antarctic fur seal inferred by STRUCTURE analysis of 2,000 individuals from eight populations genotyped at 39 microsatellite loci. Each individual is represented by a bar with the proportions of the different colours indicating the estimated membership to each of four inferred genetic populations (see Results for details). The data are plotted separately for each sampling location as indicated on the map. The map was created using ArcMap v. 10.6 https://desktop.arcgis.com/en/arcmap/.

**Figure 2:**
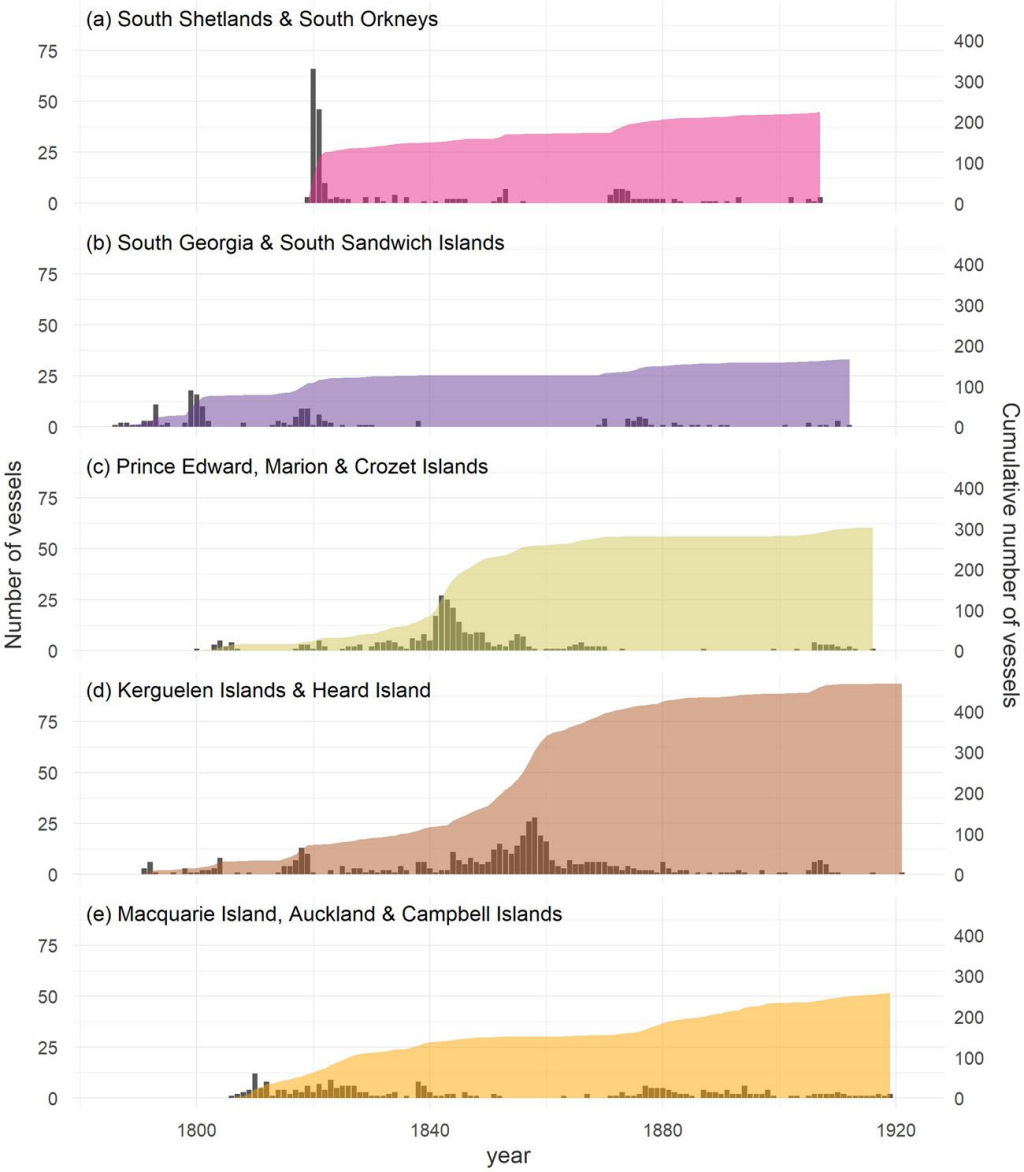
The spatial and temporal distribution of sealing effort, defined as the number of ships recorded as having visited each of the main sealing grounds. Data are replotted from Headland^29^ with the permission of the author. The islands are grouped into sealing grounds because individual ships often visited more than one island within a given geographical region; no data were available for Bouvetøya. Annual numbers of ships are shown on the left axis while the cumulative total is shown on the right axis (South Shetlands & South Orkneys = 225, South Georgia & South Sandwich Islands = 167, Prince Edward, Marion Island and Crozet Islands = 303, Kerguelen Islands and Heard Island = 469, Macquarie Island, Auckland & Campbell Islands = 259).

The Antarctic fur seal (*Arctocephalus gazella*) provides an excellent case to evaluate the genetic consequences of exploitation. This pinniped species is polygynous^24^, highly site faithful^25,26^ and has a generation time of around ten years^27^. Despite breeding on all of the major sub-Antarctic islands (Fig. 1), it was driven to the brink of extinction by the 18^th^ and 19^th^ century sealing industry^28,29^. Harvesting begun shortly after South Georgia was discovered by Captain James Cook in 1775 and ‘*reckless extermination was the only method of seal-hunting* (...) *so that the first in the field at a new sealing ground was sure of an immense booty, and late-comers as likely as not would go empty away*’^30^. Sealing reached its peak at South Georgia in 1800 when over 100,000 seals were harvested^31^. Shortly afterwards, with the discovery of the other sub-Antarctic islands, a wave of sealing spread across the entire sub-Antarctic region (Fig. 2), culminating in an estimated 1.7 million Antarctic fur seals being taken^32^.

By the turn of the 20^th^ century, Antarctic fur seals were considered all but extinct and the species was placed under legal protection. For the best part of three decades and despite several dedicated sub-Antarctic expeditions, very few individuals were sighted at the traditional breeding grounds^28,33–35^ with the exception of Bouvetøya where a small population of an uncertain fur seal species was observed in the late 1920s^36^. However, the Discovery expedition of 1936 observed a small breeding population of Antarctic fur seals with 12 pups at South Georgia^37^, which grew rapidly in the 1960s and 1970s to reach an estimated five million individuals by 1999^38^. As breeding populations were not observed at the other sub-Antarctic islands until the 1950s to the 1980s^39–44^, several authors have speculated that the former geographic distribution of this species was likely recolonized from South Georgia^28,38,45–47^. However, a number of studies have reported genetic differences among some of these populations^48–51^, pointing towards a more complex picture in which more than one relict population may have survived sealing.

In this study, we genotyped over 2,000 Antarctic fur seals at 39 microsatellite loci to investigate the genetic legacy of extreme exploitation in a marine mammal. In order to determine how the species as a whole was affected by sealing, samples from all major breeding locations were analysed to determine the most likely number of relict populations and ABC was used to reconstruct their recent demographic histories. Contrary to expectations, evidence was found for at least four genetically distinct populations, all of which experienced severe demographic declines. Nevertheless, high levels of neutral genetic variability were retained in comparison to other otariid species. Taken together, our results support the assertion that only the strongest bottlenecks lead to major losses of diversity and highlight the importance of relict populations to species recoveries and the maintenance of genetic variation.

## Results

Genetic data were generated from 2,000 Antarctic fur seals sampled from eight populations covering the circumpolar distribution of the species (see Methods for details). The 39 microsatellite loci carried between 2 and 26 alleles (mean = 11.44) and none deviated significantly from Hardy-Weinberg equilibrium in more than two out of eight sampling localities after correction for multiple tests (see Supplementary methods and results). The genotyping error rate determined by re-genotyping 96 samples was low (0.003% per allele or 0.005% per reaction).

### Population structure

Bayesian analyses of the microsatellite dataset using STRUCTURE produced consistent results across different clustering solutions (Supplementary Fig. S1). ∆*k*, which provides an indication of the uppermost hierarchical level of structure, peaked at *k* = four, while the average log likelihood of the data also gradually increased until *k* = four, after which a plateau was reached (Supplementary Fig. S2). The clustering solution for *k* = four resolved the South Shetland Islands, South Georgia and Bouvetøya as three distinct genetic clusters and Kerguelen, Heard and Macquarie Islands as a fourth cluster, while animals from Marion and Crozet Islands showed mixed membership to the third and fourth clusters (Fig. 1). Further increases in *k* only introduced additional admixture (Supplementary Fig. S1). PCA revealed a similar pattern, with Bouvetøya separating from Kerguelen, Heard and Macquarie Islands along PC1 and PC2, Marion and Crozet Islands occupying an intermediate position, and the South Shetlands and South Georgia separating along PC3 (Supplementary Fig. S3). Pairwise *F*_st_ values between populations were significant for all but two comparisons (Supplementary Table S1). Consequently, our genetic data are consistent with four relict populations comprising (i) the South Shetland Islands, (ii) South Georgia, (iii) Bouvetøya; and (iv) Kerguelen, Heard and Macquarie Islands, plus a further genetically admixed population (v) comprising Marion and Crozet Islands. Subsequent analyses therefore focused on these five populations, although similar results were obtained when sampling locations rather than genetic clusters were analysed (Supplementary Fig. S4).

### Genetic diversity

**Figure 3:**
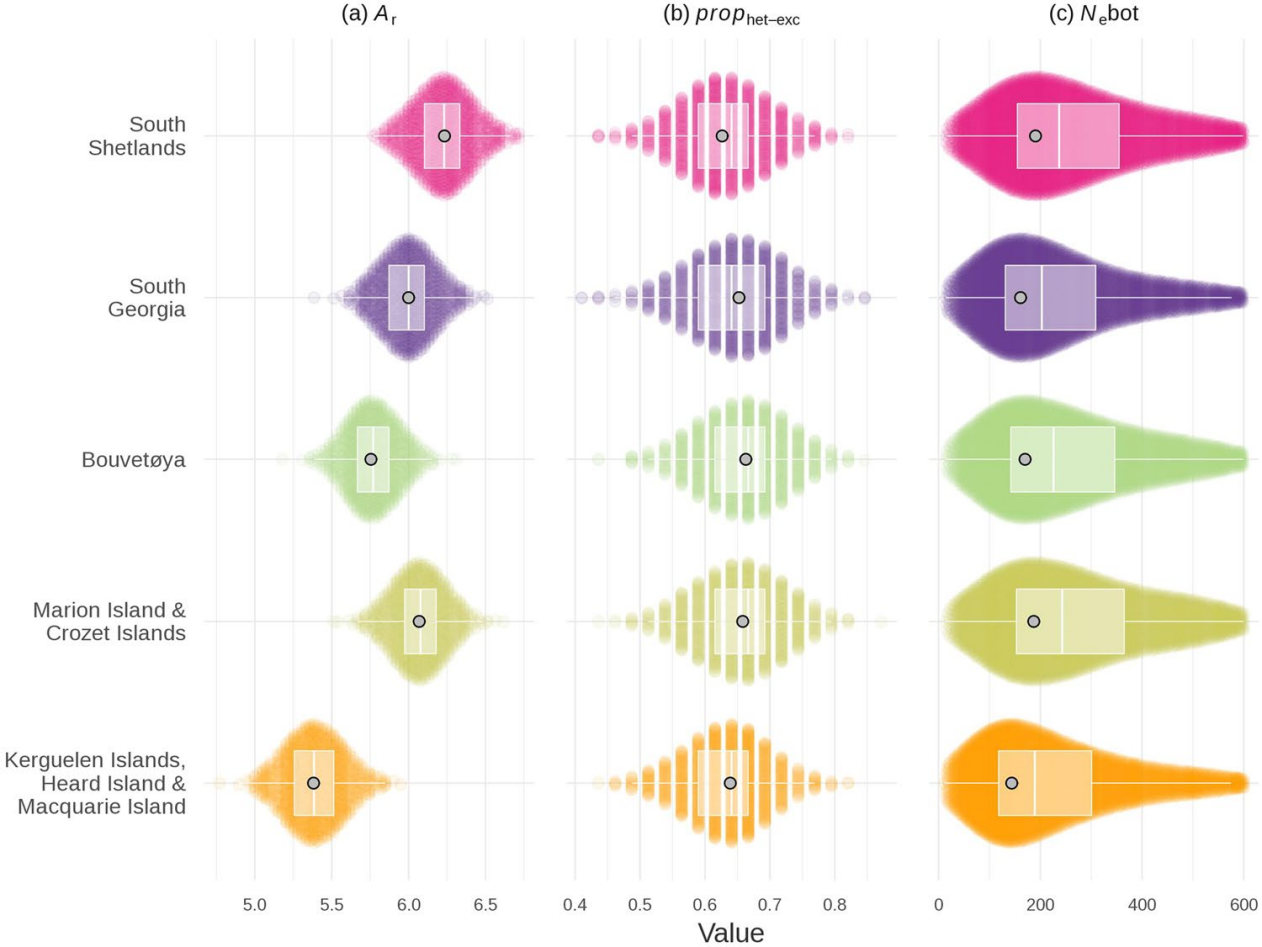
Patterns of genetic diversity and bottleneck signatures across the geographic range of the Antarctic fur seal. (**a**) Genetic diversity summarised as allelic richness (*A*_r_); (**b**) The proportion of loci in heterozygosity excess (*prop*_het-exc_) calculated for the TPM80 model; (**c**) Estimated bottleneck effective population sizes (*N*_e_bot). Data are summarised according to the five main populations identified by the STRUCTURE analysis (see Results for details). To facilitate visual comparisons among populations with different sample sizes while incorporating sampling error, we plotted 1,000 subsets of ten randomly sampled individuals per population as Sinaplots, with the exception of the sinaplot for *N*_e_bot, which shows parameter values for 5,000 accepted simulations based on 181 individuals per population. Boxplots (centre line = median, bounds of box = 25th and 75th percentiles, upper and lower whiskers = largest and lowest value but no further than 1.5 * inter-quartile range from the hinge) are superimposed with light grey points representing maximum densities. Bottleneck measures for Marion and Crozet Islands should be interpreted with caution due to admixture.

Allelic richness (*A*_r_), standardised across species as the average number of alleles per ten individuals, was high relative to other otariids, with the Antarctic fur seal being ranked fourth highest out of 13 species (Supplementary Fig. S5) for which comparable microsatellite data were available^20^. In relative terms, genetic diversity did not vary a great deal across the geographical distribution of the Antarctic fur seal (Fig. 3a, Table 1, Supplementary Table S2) with *A*_r_ only differing by around one allele per locus between the most diverse (the South Shetland Islands) and the least diverse populations (Kerguelen, Heard and Macquarie Islands).

**Table 1:** Genetic diversity and bottleneck signatures per genetic population (as determined by STRUCTURE).

Population	Sample size	*A*_r_ (SD)	*H*_o_ (SD)	M-ratio (SD)	*F*_is_ (SD)	Private Alleles	Loci_het-exc_	Sign test *p*-value	Standardized differences test *p*-value	Wilcoxon test *p*-value
South Shetlands	197	6.448 (2.555)	0.719 (0.197)	0.836 (0.185)	0.011 (0.046)	13	31	**0.004**	**<0.001**	**<0.001**
South Georgia	1042	6.235 (2.506)	0.716 (0.208)	0.813 (0.200)	0.010 (0.025)	19	34	**<0.0001**	**<0.0001**	**<0.0001**
Bouvetøya	396	5.963 (2.470)	0.703 (0.223)	0.825 (0.195)	0.014 (0.047)	8	30	**0.010**	**<0.0001**	**<0.0001**
Marion Island & Crozet Islands	184	6.279 (2.586)	0.719 (0.215)	0.830 (0.170)	0.004 (0.044)	2	31	**0.004**	**<0.001**	**<0.001**
Kerguelen Islands & Heard Island & Macquarie Island	181	5.578 (2.187)	0.688 (0.213)	0.831 (0.187)	0.006 (0.077)	7	28	0.050	**0.021**	**0.019**

Rarefied allelic richness (*A*_r_), observed heterozygosity (H_o_), M-ratio and inbreeding coefficient *F*_is_ given as means and standard deviations (SD) across 39 loci. The numbers of private alleles are summed across loci. The number of loci with heterozygosity excess (loci_het-exc_) and bottleneck test probabilities (Sign test, standardized differences tests and Wilcoxon test) are given under the two-phase model with 80% single-step mutations (TPM80) based on 1,000 iterations for each population.

### Bottleneck inference

Two complementary coalescent-based approaches were used to infer the extent to which these populations experienced recent demographic declines. First, the proportion of loci with heterozygosity-excess (*prop*_het-exc)_ provides an indicator of recent bottlenecks because rare alleles are preferentially lost during bottlenecks but their loss has little impact on heterozygosity^15^. Recent bottlenecks therefore generate a transient excess of heterozygosity relative to a population at equilibrium with an equivalent number of alleles, which can be quantified through coalescent simulation. A significant excess of heterozygosity relative to expectations was detected in all five genetic populations identified by STRUCTURE (Table 1 and Supplementary Table S1). The magnitude of *prop*_het-exc_ was also similar across populations (Fig. 3b) suggestive of more or less equally strong declines.

Second, ABC was used to select between a bottleneck and a non-bottleneck model as well as to estimate posterior distributions of the model parameters. ABC was able to distinguish between the two models, with 87% of the simulations being classified correctly under the bottleneck model and 94% of the simulations being classified correctly under the non-bottleneck model (Supplementary Fig. S6). Posterior probabilities supported the bottleneck model in all five populations and the goodness of fit to the empirical data was consistently better for the bottleneck model (Supplementary Table S3). As another indicator of model quality, posterior predictive checks^23,52^ showed that the bottleneck model was able to reproduce the relevant observed summary statistics across all populations (Supplementary Fig. S7).

As a further evaluation step, we used cross-validation to estimate prediction errors for all model parameters. Cross-validation in ABC uses randomly selected simulations and treats them as pseudo-observed datasets. For each of these simulations, the ABC model was used to estimate a set of summery statistics. Because we also know the true summary statistics, we can calculate the prediction error as ${E}_{pred}={\Sigma }_{i}{({\bar{\theta }}_{i}-{\theta }_{i})}^{2}/Var({\theta }_{i})$, where ${\bar{\theta }}_{i}$ is the estimated parameter value and *θ*_*i*_ is the true parameter value of the i^th^ simulated data set. If the estimated values are closer to the true values than expected by chance (i.e. they contain information about the true values), the prediction error will be below one. Under the bottleneck model, the prediction error was 0.457 for the bottleneck effective population size (*N*_e_bot), indicating that posterior estimates for this parameter contain information about the underlying true values (Supplementary Fig. S8). Although in principle the posterior estimates for *N*_e_hist also had an acceptable prediction error (0.611), a plot of the cross-validation results (Supplementary Fig. S8) revealed a systematic underestimation of the true values, which is why the posteriors should be interpreted with caution. By contrast, prediction errors for the other parameters were substantially higher (Supplementary Table S4), with visual inspection of the cross-validation results revealing a large amount of scatter and a poor fit of the predicted to the true values. Consequently, subsequent analyses focused on the posteriors for *N*_e_bot, while the *N*_e_hist estimates are also shown in Supplementary Fig. S9.

The *N*_e_bot estimates were similar in magnitude across populations (Fig. 3c), which is again suggestive of recent bottlenecks of comparable strength. However, the posterior distribution of *N*_e_bot was skewed towards slightly smaller values (maximum density ~150) in the eastern population comprising Kerguelen, Heard and Macquarie Islands, whereas maximum densities were closer to ~200 elsewhere. Similar results were obtained when the sampling locations were analysed separately (Supplementary Fig. S4). The *N*_e_hist posterior estimates were very broad (Fig. S9), suggesting that historical effective population sizes ranging between a few thousand and tens-of-thousands of individuals could have led to similar observed genetic diversities under the bottleneck model.

### Expected loss of genetic diversity

**Figure 4:**
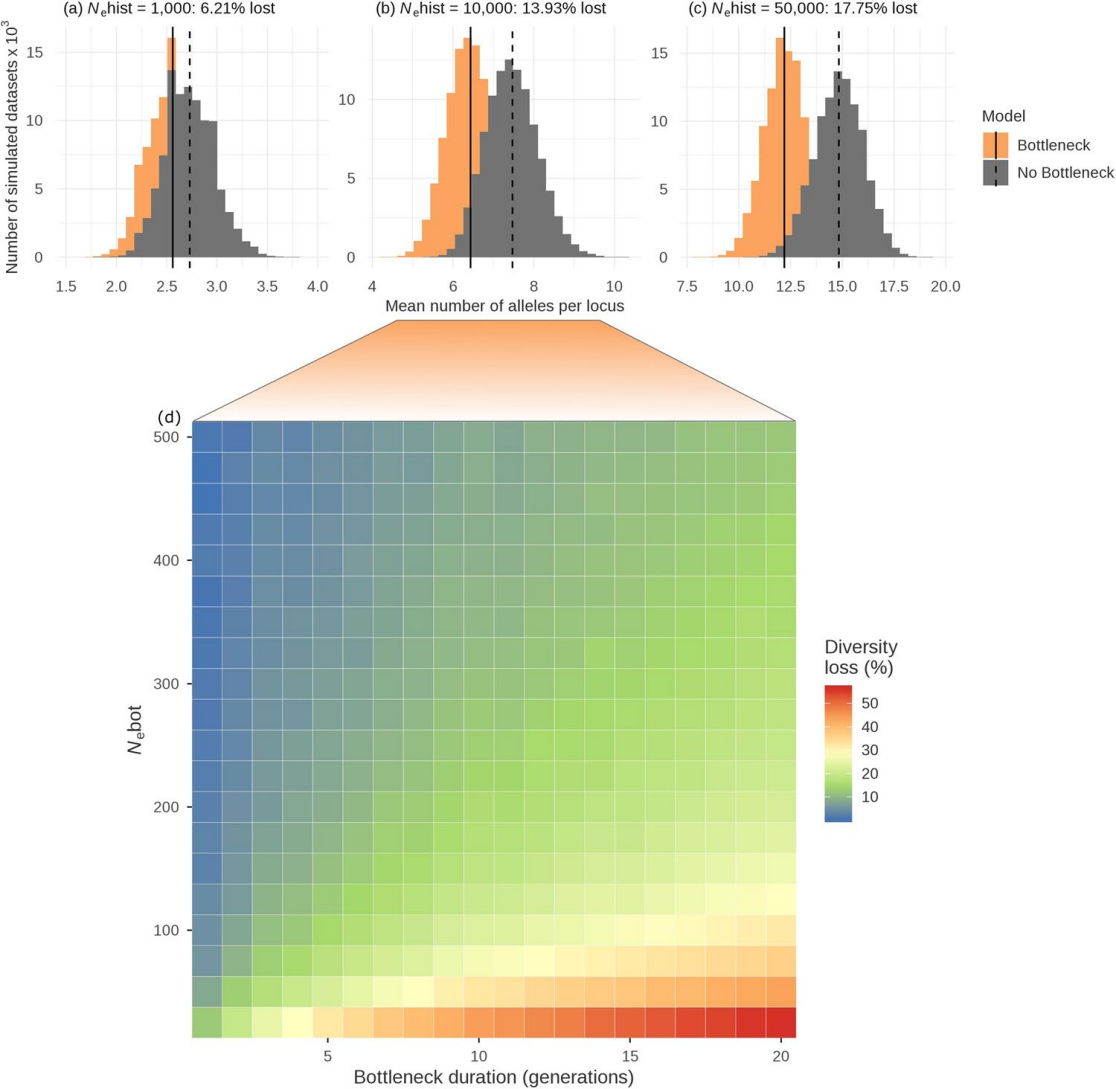
The expected loss of genetic diversity caused by recent bottlenecks. Panels (a–c) show the distributions of mean allele numbers across loci obtained from 100,000 neutral coalescent simulations of a bottleneck to an *N*_e_bot of 200 for ten generations (orange) and a constant population size scenario (grey) with three different pre-bottleneck effective population sizes (*N*_e_hist). Panel (d) further explores the effects of bottleneck duration and strength on the expected loss of allelic diversity under the scenario shown in panel (b) as described in the Methods.

The expected loss of neutral genetic diversity caused by a bottleneck with an effective size *N*_e_bot of 200 lasting for ten generations was evaluated using coalescent simulations in fastsimcoal^53^. As the long-term *N*_e_ prior to the bottleneck will affect overall levels of diversity, three scenarios were analysed with *N*_e_hist simulated as 1,000, 10,000 and 50,000 respectively. Despite a severe and relatively long-lasting simulated bottleneck, the expected loss of microsatellite alleles was small, ranging from 6% with an *N*_e_hist of 1,000 to 18% with an *N*_e_hist of 50,000 (Fig. 4). Under the scenario that produced levels of genetic diversity most similar to the empirical values (*N*_e_hist = 10,000) only around 14% of alleles were lost. Furthermore, simulated bottlenecks of varying strength and duration, again with an *N*_e_hist of 10,000, suggest that only a combination of very strong (i.e. *N*_e_bot < 50) and long (i.e. 15–20 generations) bottlenecks would result in genetic diversity losses in excess of 50%. Consequently, the Antarctic fur seal is unlikely to have lost a substantial proportion of neutral genetic diversity despite an inferred steep decline in *N*_e_ due to overhunting.

## Discussion

We investigated the genetic consequences of historical commercial exploitation in the Antarctic fur seal. Information on the number of sealing vessels recorded as having visited each of the main sealing grounds allowed us both to formulate realistic priors for our ABC analysis and to interpret our results in the light of historical evidence. An innovation of this study was to use a comparative approach to shed light on the genetic diversity and recent demographic histories of multiple populations within a single species.

Consistent with previous studies of Antarctic fur seals, we found evidence for strong population structure^47–50,54^. However, our larger sample size of individuals and exhaustive geographical coverage yields a more comprehensive global picture. On the one hand, the discovery of four genetically distinct clusters builds upon recent studies suggesting that relict populations likely persisted in localities other than South Georgia^49,50^. This is in contrast to the previously held belief that Antarctic fur seals only survived in one relict population off South Georgia^28,38,45,46^ and suggests that, at least in some regards, the species as a whole may have been more resilient to commercial exploitation than previously believed. Nevertheless, our data also suggest that at least one and possibly several populations may have gone locally extinct. In particular, Macquarie, Heard and Kerguelen Islands showed little in the way of population structure, consistent with the suggestion that this species was locally extirpated at Macquarie and Heard Islands, leaving these sites to be subsequently recolonised by individuals mainly from the Kerguelen Archipelago^47,55^. In addition, a strong signal of admixture at Marion and Crozet Islands implies that these two populations may have either gone extinct or been reduced to sufficiently small numbers for the local gene pool to be swamped by emigrants from nearby Bouvetøya and the Kerguelen Islands. This is supported by anecdotal evidence suggesting that Antarctic fur seals were not present after sealing ceased at Marion Island in the early 1930s^56^.

Two approaches were used to infer the strength of recent population declines. Heterozygosity excess provides a measure of the *relative* strength of population decline, whereas our ABC analysis specifically tested for an *absolute* reduction in *N*_e_ (i.e. to below 600). By focusing on time priors that reflected the known sealing history, the ABC analysis also evaluated a clear hypothesis‒that 18^th^ and 19^th^ century commercial sealing resulted in strong bottlenecks. Although in principle, similar patterns of genetic diversity might be produced by more ancient bottlenecks, these are unlikely to be detected reliably using microsatellite data when subsequent recovery occurred^57^. This is in keeping with a previous study in which a simple model incorporating a recent bottleneck was better supported than a more complex model that also included a small population size during the last glacial maximum followed by expansion^20^.

In practice, heterozygosity excess and ABC produced consistent results, with all of the populations identified by STRUCTURE showing a significant excess of heterozygosity as well as support for recent demographic declines in the ABC analysis. Similar results were also obtained after analyzing each of the sampling localities separately, although Heard Island failed to reach significance in the heterozygosity excess test, possibly due to our relatively small sample size of individuals from this locality. *N*_e_bot estimates from the ABC analysis were also quite similar across populations, although the mode of the posterior distribution was slightly lower for the easternmost population. This subtle difference in inferred bottleneck intensity is a reflection of the lower genetic diversity of the Kerguelen Islands, Heard and Macquarie Island populations. It is furthermore consistent with the historical sealing data (Fig. 2), which show that sealing effort was higher in these areas, with Kerguelen and Heard Islands in particular having been visited by more ships in total than any of the other main sealing grounds.

Overall, however, we were struck by the similarity of the *N*_e_bot estimates, whose maximal densities consistently fell within the range of around 150 to 200. This provides a good indication that census population sizes on all of the islands may have been similarly diminished, although the relationship between *N*_e_ and census size has to be interpreted with caution^58^. Moreover, mammalian effective population sizes are usually around 2‒3 times smaller than census population sizes^59^ and this ratio may be even greater in fur seals due to their strongly polygynous mating system^24^. Our results therefore suggest that the global population of Antarctic fur seals that survived sealing was considerably larger than implied by historical accounts and may have numbered in the thousands.

Several factors may play a role in explaining our results. First, standard economic theory predicts that exploitation will not necessarily lead to extinction due to the escalating costs of harvesting increasingly depleted populations^1,60^. Consistent with this explanation, historical sealing data show a geographically concordant pattern of early intense exploitation followed by greatly diminished sealing effort during the mid to late 19^th^ century (Fig. 2). This reduction in sealing effort coincides with a crash in the price of fur seal pelts in America and China during the 1850s, which would have substantially decreased profitability^61^. Consequently, the available historical evidence suggests that sealing probably ceased to become economically viable around the time when the main sealing grounds had been largely depleted of animals.

Having said this, historical accounts suggest that the sealers were extremely thorough in the pursuit of their quarry. Not only did their high mobility allow them to quickly discover and exploit new hunting grounds^62^, but it was also commonplace for sealing gangs to be left ashore for extended periods to opportunistically harvest any animals that remained on land^28,63^. It therefore seems unlikely that appreciable numbers of individuals could have survived ashore at the main breeding grounds. This may explain the rarity of sightings of this species during the first few decades of the 20^th^ century^28,33–35^ and the fact that some breeding populations were not re-established until as late as the 1980s^39–44^.

It is conceivable that some juveniles, subadults and non-breeders could have escaped the sealers by not coming ashore. However, if this were the case, relict populations should have survived at all of the breeding grounds, whereas our data, together with recent studies and historical accounts, suggest that that Antarctic fur seals may have been locally extirpated at Macquarie, Heard, Crozet and Marion Islands^47,50,51^. We therefore speculate that tiny remnants of once much larger breeding populations may have persisted in a handful of particularly inaccessible locations that were rarely if ever visited by sealers. Candidates for sites that may have sheltered these relict populations include the Willis Islands off the coast of South Georgia^28^, the San Telmo Islets in the South Shetlands^49^ Larsøya and Kapp Norvegia at Bouvetøya^36^ and any one of a multitude of small islands in the Kerguelen Archipelago^48^.

This study contributes towards wider debate about the factors shaping genetic diversity and its importance for conservation. In particular, many studies have reported links between known historical bottlenecks and low levels of genetic diversity^64–67^. However, the implied loss of diversity is often greater than predictions from population genetic theory^68,69^, suggesting that other mechanisms such as selective sweeps could be involved^70^. Additionally, the general perception that bottlenecked populations have low genetic variability may also have been influenced by publication bias^68^. In the current study, it was not possible to directly quantify the amount of genetic diversity lost as a result of sealing due to a lack of historical samples. However, coalescent simulations provided an expectation of the likely magnitude of genetic erosion given the strength of the inferred demographic declines. These simulations suggest that a recent decline from an historical *N*_e_ of 10,000 to an *N*_e_bot of 200 for ten generations is unlikely to have resulted in the loss of more than around 15% of alleles. Consequently, our study supports the argument of Amos and Balmford^68^ that even extreme recent bottlenecks need not necessarily lead to major losses of genetic diversity. This may further help to explain why several other pinniped species including northern, Australian and Juan Fernandez fur seals also retained high levels of genetic diversity despite having been harvested in their tens of thousands to millions^71–73^.

To conclude, demographic reconstruction of an entire species provided insights into the resilience of a marine mammal that was hunted in very large numbers. Our study suggests that, although local extinctions took place, Antarctic fur seals persisted in sufficient numbers in several relict populations to retain a large proportion of the species᾿ pre-sealing genetic diversity. The extreme geographical isolation of some small breeding sites in addition to diminishing economic incentives for sealing likely saved the Antarctic fur seal from extinction. The subsequent recovery of this species is a testament both to its resilience and to the success of protection measures in the 20^th^ century.

## Methods

### Tissue sampling and microsatellite genotyping

Samples were collected from a total of 2,064 Antarctic fur seal individuals from eight different breeding locations (Fig. 1, for GPS coordinates see Supplementary Table S1). Skin samples were taken from the interdigital margin of the foreflipper with piglet ear notching pliers^74^ and stored at −20°C in 20% dimethyl sulphoxide saturated with sodium chloride. DNA was extracted using a chloroform/isoamylalcohol extraction protocol^75^ and genotyped at 39 microsatellite loci as described in the Supplementary methods.

Because Antarctic fur seals are known to hybridise with Subantarctic fur seals (*Arctocephalus tropicalis*), we additionally sampled 91 pups from a reference population of *A. tropicalis* at Macquarie Island in order to check the full *A. gazella* dataset for individuals with a high proportion of *A. tropicalis* ancestry (see Supplementary methods and results). We found that 49 of the Antarctic fur seal individuals were inadvertently pure *A. tropicalis*, while five of the *A. tropicalis* reference individuals were hybrids (see Figs. S11 and S12). A further 15 Antarctic fur seal individuals were also identified as hybrids. After having removed all pure *A. tropicalis* individuals as well as the hybrids and any additional individuals that failed to genotype at more than four loci from the dataset, we were left with a total of 2,000 Antarctic fur seal individuals, comprising 197 from the South Shetland Islands, 1,042 from South Georgia, 396 from Bouvetøya, 166 from Marion Island, 18 from Crozet Islands, 51 from Kerguelen Islands, 22 from Heard Island and 108 from Macquarie Island.

### Population structure and genetic diversity

For the full dataset of 2,000 Antarctic fur seals, we evaluated whether population structure could be detected without prior knowledge of sampling locations using STRUCTURE version 2.3.3^76^. This program distributes individual genotypes into *k* clusters by subdividing the dataset in a way that maximizes Hardy–Weinberg and linkage equilibrium within the resulting clusters. The membership of each individual to a given cluster is then estimated as *q*, which varies between 0 and 1, with the latter indicating full cluster membership. STRUCTURE was run using ParallelStructure^77^ with the following parameters: 500,000 burn-in length, 1,000,000 MCMC replications, 1‒10 assumed clusters (*k*) and 10 iterations for each *k*. The most likely number of clusters was evaluated using the maximal average value of Ln *P*(*D*) as well as the Evanno method^78^. Individual admixture proportions were integrated over all simulations using pophelper^79^. For comparison, we also implemented principle component analysis (PCA) using the R-package adegenet^80^. Genetic diversity was quantified as allelic richness (*A*_r_) within hierfstat^81^. Observed heterozygosity (*H*_o_) was calculated with adegenet^80^, the M-ratio was calculated with StrataG^82^, the inbreeding coefficient *F*_is_ was calculated with diveRsity^83^ and the number of private alleles was calculated with poppr^84^. Pairwise *F*_st_ values together with their 95% confidence intervals were obtained using hierfstat^81^.

### Bottleneck analyses

We used two coalescent-based approaches to infer recent demographic histories. First, we quantified the proportion of loci in heterozygosity-excess using the program BOTTLENECK v. 1.2.02^85^. This analysis was based on the two-phase model with 80% single-step mutations (TPM80), consistent with recent estimates from multiple pinniped species^20^. A total of 1,000 iterations were run for each population and all other parameters were left at default values. Statistical significance was evaluated using sign, standardized differences and Wilcoxon signed ranks tests.

Second, we tested for signatures of recent population bottlenecks using a coalescent-based approximate Bayesian computation (ABC) framework. Support was evaluated for two alternative demographic models (Supplementary Fig. S10). Briefly, to evaluate whether 18^th^ to 20^th^ century commercial exploitation caused severe demographic reductions, we defined a “bottleneck model”, which incorporated a strong decline in population size within strictly bound time priors that fit the time period hunting took place. The priors for pre- and post-bottleneck effective population size were drawn independently from the same distribution to allow the model not only to incorporate a bottleneck, but also longer-term reductions or expansions within realistic bounds. For comparison, we defined a “non-bottleneck model” which was identical to the bottleneck model with the exception of a bottleneck.

For both models, priors for current effective populations size (*N*_e_) and historical pre-bottleneck effective population size (*N*_e_hist) were drawn from a log-normal distribution in order to encompass a wide range of values while mainly sampling from the most likely ones. We specified *N*_e_ and *N*_e_hist ~ lognorm[logmean = 10.5, logsd = 1], which concentrated around 90% of sampling in the range between 3,000 and 15,000 while also occasionally simulating much larger *N*_e_ values. This prior range encompassed the estimated current census population sizes across all islands, i.e. from 3,600 at Marion Island^86^ to 5 × 10^6^ at South Georgia^38^. For the bottleneck model, the effective population size during the bottleneck (*N*_e_bot) was drawn from a uniform distribution between 1 and 600 (*N*_e_bot ~ U[1, 600]). Start and end time for the bottleneck were defined by *t*_bot_start and *t*_bot_end and drawn from uniform distributions between ten and 40 (*t*_bot_start ~U[10, 40]) and one and 20 (*t*_bot_end~ U[1, 20]) generations ago respectively. This places the bottleneck in the last one to four centuries, as the generation time of the Antarctic fur seal is estimated at around nine years^87^. For the neutral model, the time parameter corresponding to the historical population size (*t*_hist_) was drawn from a uniform distribution ranging between 10 and 40 generations ago (*t*_hist_ ~U[10, 40]). The microsatellite mutation rate (*µ*) was drawn from a uniform prior with *µ* ~ U[10^−5^, 10^−4^]. A stepwise mutation model was used with the geometric parameter GSMpar reflecting the proportion of multistep mutations, uniformly distributed from GSMpar ~U[0, 0.3].

Genetic data were simulated under each demographic model for 181 individuals, reflecting the smallest genetic population (as determined by STRUCTURE) in the empirical dataset, and 39 microsatellites using fastsimcoal2^17^ within the R-package strataG^82^. This resulted in a total of 2 × 10^7^ datasets. We then calculated three summary statistics (as means across all loci) for the simulated data: (1) allelic richness; (2) the proportion of low frequency (<5%) alleles; and (3) the M-ratio^88^, and retained the 5,000 simulations with summary statistics closest to the empirical data using a tolerance threshold of 5 × 10^−4^.

To evaluate model specification and fit, we implemented leave-one-out cross-validation using the *cv4postpr* function of the abc R-package^89^. Here, simulations are selected at random and their summary statistics are used as pseudo-observed data. The remaining simulations are then used to classify the data into the bottleneck or the neutral model. If ABC is capable of distinguishing between the two models, a large posterior probability will be assigned to the same model under which the pseudo-observed data were generated. This procedure was repeated 100 times and the posterior probabilities for a given model were averaged to derive the rate of misclassification. Furthermore, posterior probabilities were calculated for each model using the rejection method of the *postpr* function and the fit of the preferred model to the empirical data was evaluated using a formal hypothesis testing procedure implemented by the *gfit* function of the abc R-package.

Next, we tested the accuracy of the ABC parameter estimates using leave-one-out cross validation in the *cv4abc* function. For a randomly selected pseudo-observed dataset, parameters were estimated using all remaining simulations via ABC using the rejection method. This procedure was repeated 1,000 times and the mean prediction error was calculated. The smaller the prediction error, the better the fit to the true parameter value. After performing the four checks, we constructed posterior distributions of the parameters using a simple rejection algorithm with the *abc* function of the abc package.

To provide a measure of the fit of the preferred models to the empirical data, we conducted posterior predictive checks^23,52^. After generating posterior distributions for each parameter, 1,000 multivariate parameters were sampled from their respective posterior distributions and used to simulate summary statistics *a posteriori* based on the preferred model. Those summary statistics were then plotted as histograms and superimposed over the observed summary statistics^23^.

### Expected loss of genetic diversity

Finally, we used neutral coalescent simulations in fastsimcoal2^53^ to estimate the expected loss of microsatellite alleles for a bottleneck effective population size (*N*_e_Bot) of 200 lasting for ten generations. As the historical effective population size *N*_e_hist is unknown but will be an important determinant of the overall level of genetic diversity, we simulated three different scenarios with *N*_e_hist values of 1,000, 10,000 and 50,000. The post-bottleneck *N*_e_ is unlikely to have a major effect on diversity, as only a few generations have passed since the bottleneck, and was thus simulated equal to the *N*_e_hist within each scenario. All other model parameters were the same as in the models above. We then compared the resulting allelic richness to the allelic richness generated in equivalent models with constant effective population sizes of 1,000, 10,000 and 50,000 respectively to estimate the proportional loss of alleles due to the bottleneck. To account for uncertainty in building coalescent trees, we simulated 100,000 datasets under both the bottleneck and constant size model and treated the difference in mean allelic richness between the distributions as the average loss of alleles. To further explore the effects of varying bottleneck strength and duration, we conducted a final round of simulations based on the scenario that provided the best fit to the empirical data (*N*_e_hist = 10,000, see Results). Specifically, we quantified the expected loss of alleles for *N*_e_bot ranging 25 to 600 in steps of 25 and for bottleneck duration ranging from one to 20 generations in steps of one. As hunting ceased by the turn of the 20^th^ century, we fixed *t*_bot_end at ten generations ago. 1,000 simulations were conducted for each combination of parameter values.

### Animal ethics

Samples were collected from the South Shetlands under Marine Mammal Protection Act Permit no. 774-1847-04 granted by the Office of Protected Resources, National Marine Fisheries Service. Sampling and procedures used on South Georgia were approved by the British Antarctic Survey Animal Welfare and Ethics Review Body (reference no. PEA6) and collected as part of the Polar Science for Planet Earth programme of the British Antarctic Survey. Samples from Bouvetøya were obtained under Permit no. 7001 issued by the Norwegian Department of Plants, Fish, Animals and Food. Samples were collected on Marion Island, Crozet Islands, Kerguelen Islands, Heard Island and Macquarie Island with approval from the Animal Ethics Committee of the Faculty of Veterinary Science, University of Pretoria, South Africa (PN 859; EC077-15), the Prince Edward Islands Management Committee and Department of Environmental Affairs, the Territory of Heard Island and McDonald Islands Environment Protection and Management Ordinance 1987 (Permit no. 00/18) and the Parks and Wildlife Service, Tasmania (Scientific Collecting Permit no. FA 99167). All sampling and procedures were performed in accordance with the relevant guidelines and regulations.

## Supplementary information

Supplementary information.

## Data Availability

All of the raw data^90^ are available via the Zenodo repository, doi:10.5281/zenodo.3585717.

## Code availability

All data wrangling steps and statistical analyses except for the heterozygosity-excess tests were implemented in R. The complete documented analysis pipeline is available via our GitHub repository: https://github.com/apaijmans/AFS_genetic_legacy.
