# Publisher Correction: The genetic legacy of extreme exploitation in a polar vertebrate

**DOI:** 10.1038/s41598-020-64763-1

**Published:** 2020-05-12

**Authors:** Anneke J. Paijmans, Martin A. Stoffel, Marthán N. Bester, Alison C. Cleary, P. J. Nico De Bruyn, Jaume Forcada, Michael E. Goebel, Simon D. Goldsworthy, Christophe Guinet, Christian Lydersen, Kit M. Kovacs, Andrew Lowther, Joseph I. Hoffman

**Affiliations:** 1 Department of Animal Behaviour, Bielefeld University, 33501 Bielefeld, Germany; 2 Department of Zoology and Entomology, Mammal Research Institute, University of Pretoria, Private Bag X20, Hatfield, 0028 South Africa; 3 Norwegian Polar Institute, Fram Centre, 9296 Tromsø, Norway; 4 Department of Natural Sciences, University of Agder, 4630 Kristiansand, Norway; 5 British Antarctic Survey, High Cross, Madingley Road, Cambridge, CB3 OET UK; 6 Antarctic Ecosystem Research Division, Southwest Fisheries Science Center, National Marine Fisheries, National Oceanographic and Atmospheric Administration, 8901 La Jolla Shores Drive, La Jolla, CA 92037 USA; 7 South Australian Research and Development Institute, 2 Hamra Avenue, West Beach, South Australia 5024 Australia; 8 School of Biological Sciences, The University of Adelaide, Adelaide, South Australia 5005 Australia; 9 Centre d’Etudes Biologiques de Chizé (CEBC), CNRS and Université de La Rochelle - UMR 7372, 79360 Villiers en Bois, France

Correction to: *Scientific Reports* 10.1038/s41598-020-61560-8, published online 20 March 2020

The original version of this Article contained errors.

As a result of a typesetting error, in the original PDF version of the Article Affiliation 5 was incorrectly listed as ‘Present address: Department of Natural Sciences, University of Agder, 4630, Kristiansand, Norway.’. The correct affiliation is listed below:

Department of Natural Sciences, University of Agder, 4630, Kristiansand, Norway.

Additionally, in the original HTML version of this Article, Michael E. Goebel was incorrectly affiliated with ‘South Australian Research and Development Institute, 2 Hamra Avenue, West Beach, South Australia, 5024, Australia’. The correct affiliations are listed below:

Antarctic Ecosystem Research Division, Southwest Fisheries Science Center, National Marine Fisheries, National Oceanographic and Atmospheric Administration, 8901 La Jolla Shores Drive, La Jolla, CA, 92037, USA.

Present address: Institute of Marine Science, University of California Santa Cruz, 1156 High Street, Santa Cruz, CA, 95064, USA.

These errors have now been corrected in the HTML and PDF versions of this Article.

