# Supplementary material for: The genetic legacy of extreme exploitation in a polar vertebrate
Source: Sci Rep. 2020 Mar 20;10:5089. doi: 10.1038/s41598-020-61560-8 (PMC7083876; doi:10.1038/s41598-020-61560-8)
Supplement: Supplementary file 1 — Supplementary information. [file 41598_2020_61560_MOESM1_ESM.pdf]

Supplementary Information

The genetic legacy of extreme exploitation in a polar vertebrate

Anneke J. Paijmans, Martin A. Stoffel, Marthán N. Bester, Alison C. Cleary, P. J. Nico De Bruyn, Jaume Forcada, Mike E. Goebel, Simon D. Goldsworthy, Christophe Guinet, Christian Lydersen, Kit M. Kovacs, Andrew Lowther & Joseph I. Hoffman

Table of contents

SI figures and tables **Page 2**

SI methods and results– microsatellite genotyping and hybrid analyses **Page 15**

SI figures and tables

Fig. S1. STRUCTURE results for $k = 2$ to $k = 8$ for the full dataset of 2,000 *A. gazella* individuals (excluding hybrids and *A. tropicalis*) based on 39 microsatellites. Each individual is represented by a vertical bar with the proportion of the colouring representing the probability of belonging to each cluster. Individuals are grouped per sampling location, from left to right: South Shetland Islands (SSI), South Georgia (SG), Bouvetøya (BI), Marion Island (MAR), Crozet Islands (CI), Kerguelen Islands (KI), Heard Island (HI) and Macquarie Island (MAC).

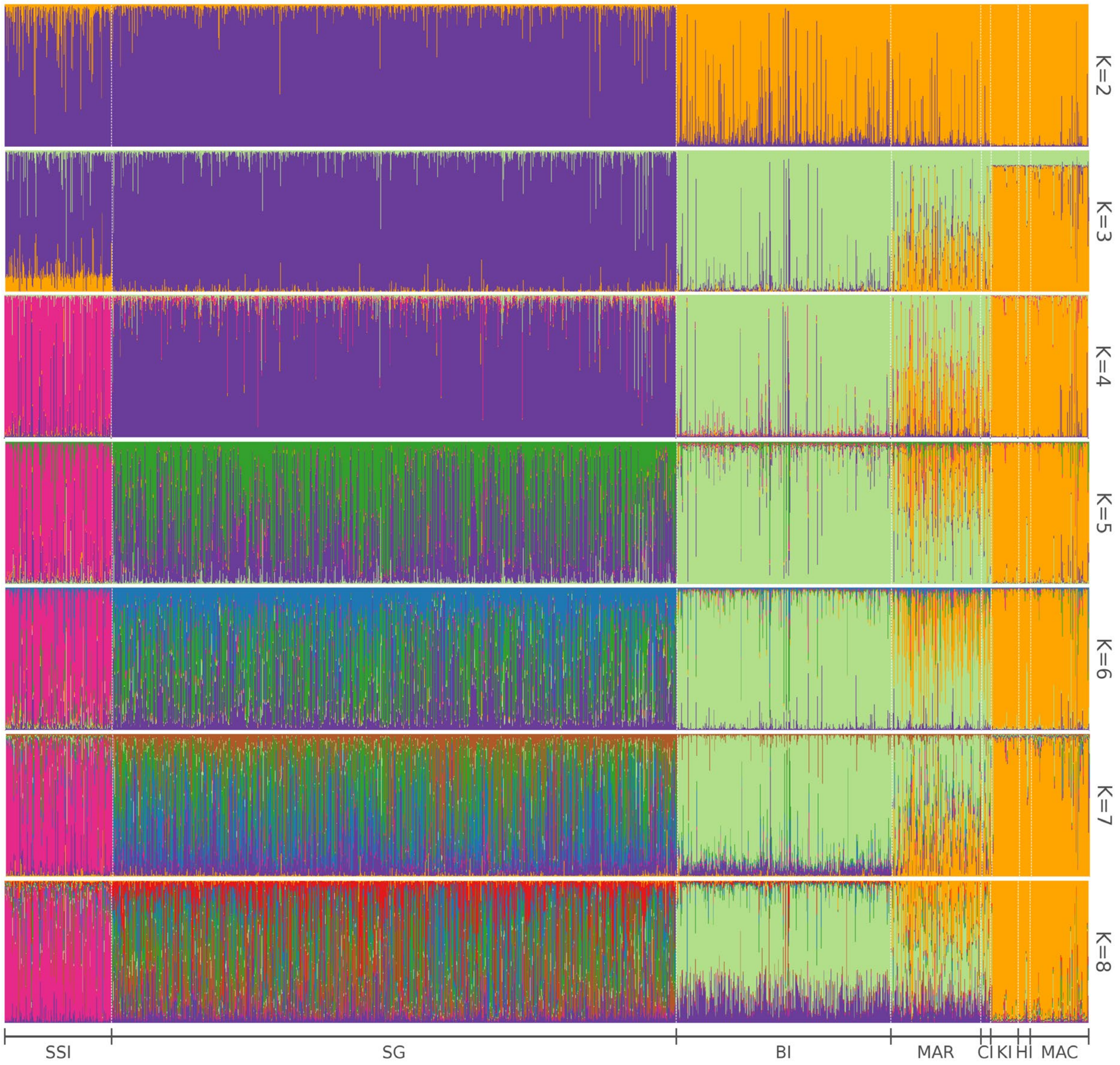

Fig. S2. (a) Delta *k* and (b) Ln Pr(x | *k*) values with standard errors calculated for 10 replicate runs of STRUCTURE for *k* = 1 to *k* = 10 using the full *A. gazella* dataset.

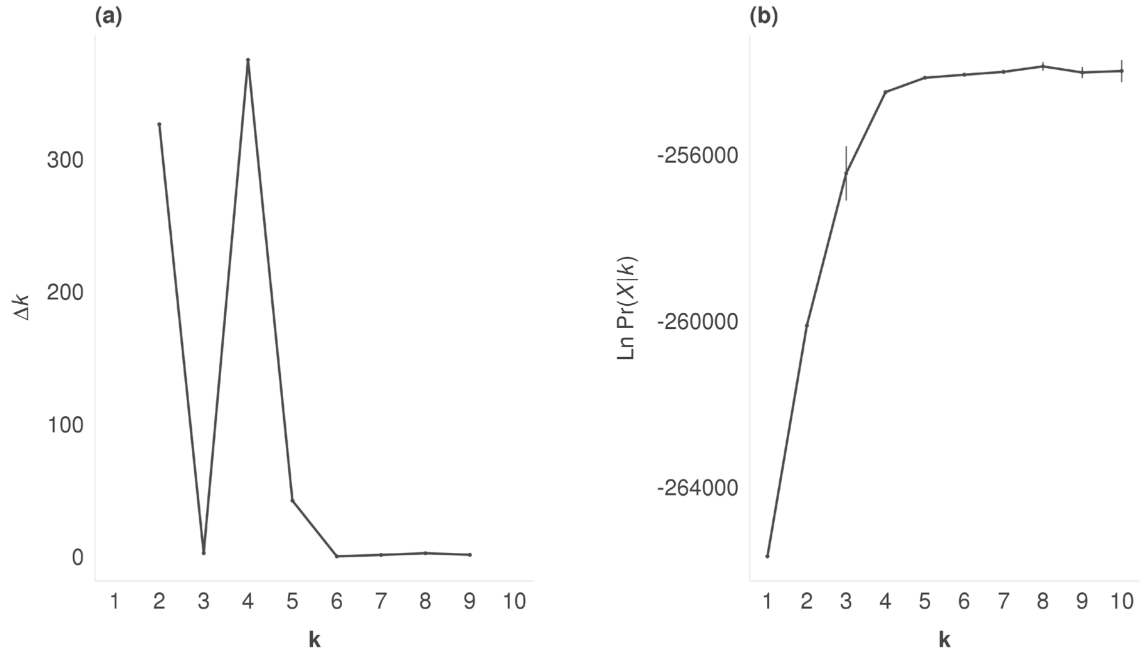

Fig. S3. Results of the PCA of the full *A. gazella* dataset. Points represent individual variation in (a) principal components one and two; and (b) principal components one and three. Colours represent sampling locations as indicated in the legend.

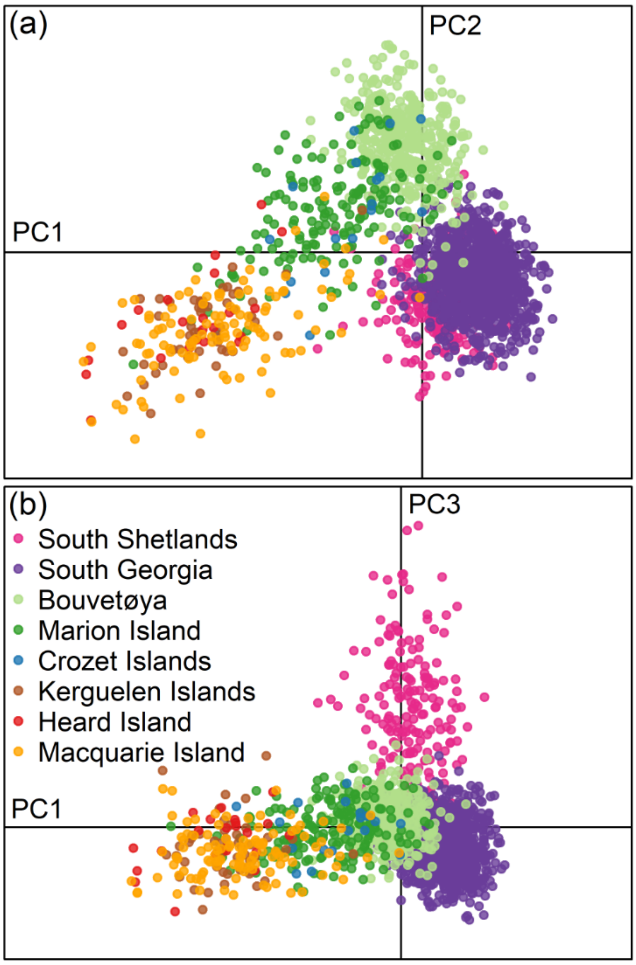

Fig. S4. Patterns of genetic diversity and bottleneck signatures across the geographical range of the Antarctic fur seal. (a) Genetic diversity summarised as allelic richness (A_r); (b) The proportion of loci in heterozygosity excess ($prop_{het\text{-}exc}$) calculated for the TPM80 model; (c) Estimated bottleneck effective population sizes ($N_e$bot). Data are summarised according to the eight sampling locations. To facilitate visual comparisons among populations with different sample sizes, while also incorporating sampling error, 1,000 subsets of ten randomly sampled individuals per population were plotted as Sinaplots, with the exception of the sinaplot for $N_e$bot, which shows parameter values for 5,000 accepted simulations based on 18 individuals per population. Boxplots (centre line = median, bounds of box = 25th and 75th percentiles, upper and lower whiskers = largest and lowest value but no further than 1.5 * inter-quartile range from the hinge) are superimposed with light grey points representing maximum densities. Bottleneck measures for Marion and Crozet islands should be interpreted with caution due to admixture.

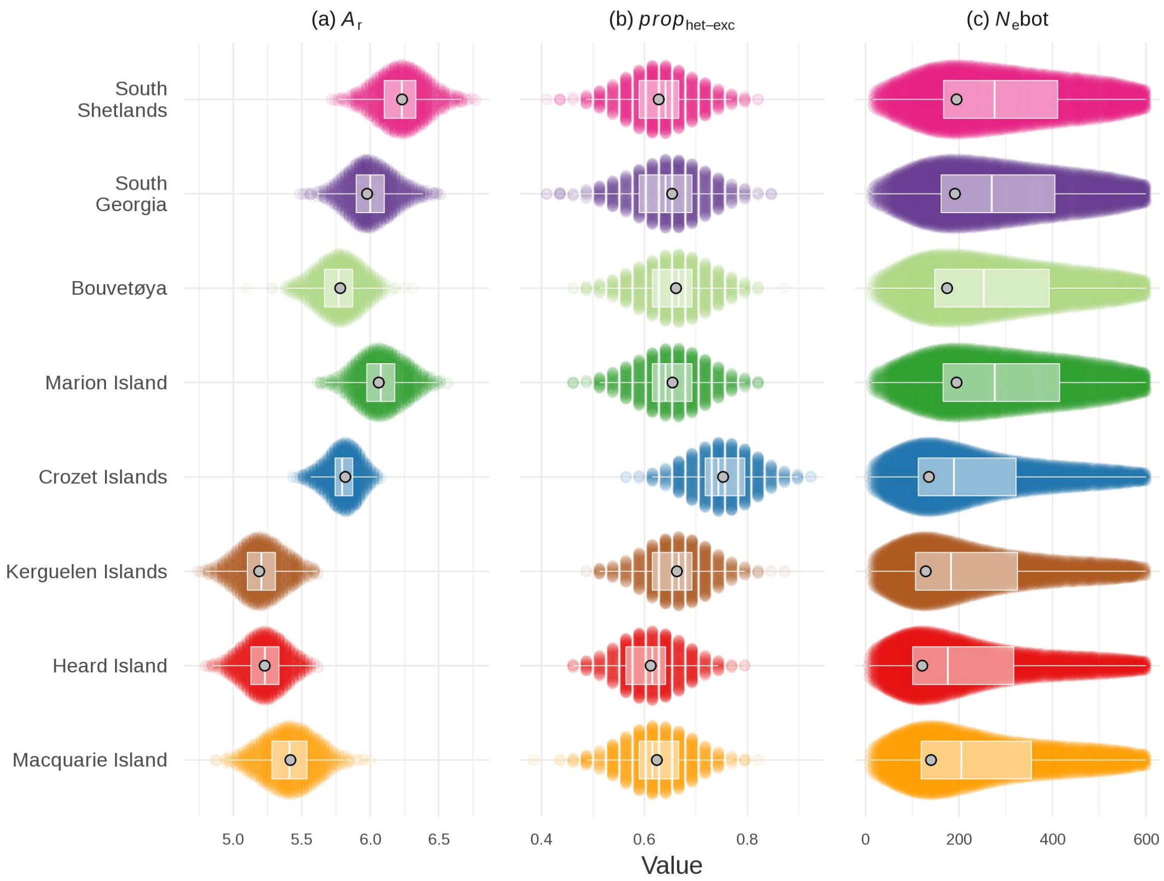

Fig S5. Genetic diversity of Antarctic fur seals relative to other otariid species. To facilitate visual comparisons among species with different sample sizes while also incorporating sampling error, we plotted microsatellite allelic richness (A_r) for 1,000 subsets of ten randomly sampled individuals per population as Sinaplots. Boxplots (centre line = median, bounds of box = 25th and 75th percentiles, upper and lower whiskers = largest and lowest value but no further than 1.5 * inter-quartile range from the hinge) are superimposed with light grey points representing maximum densities. Species are presented in ascending order of median A_r with the Antarctic fur seal highlighted in orange.

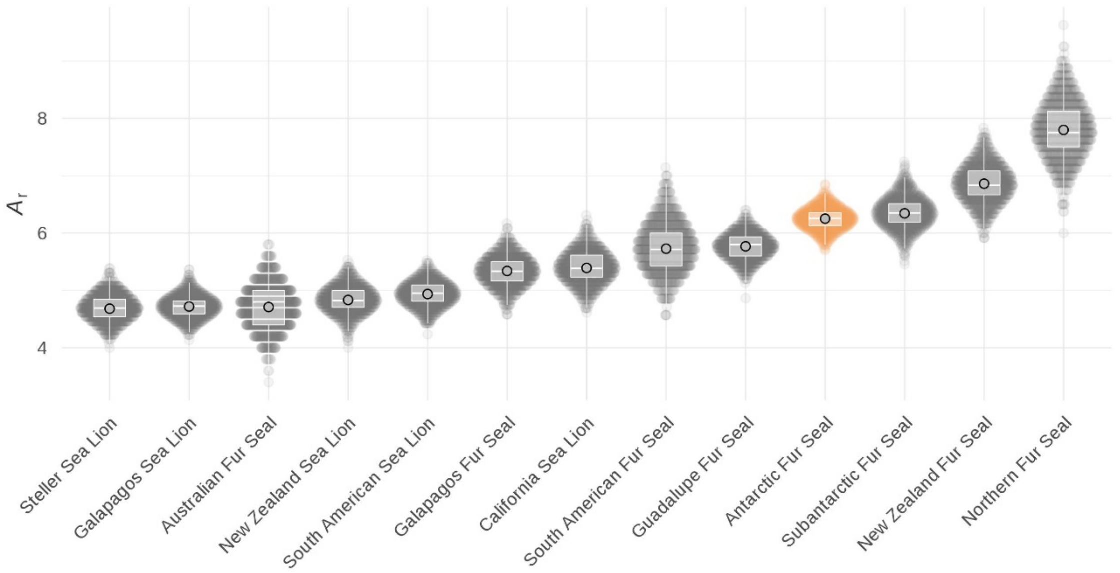

Fig. S6. Confusion matrix showing misclassification rate estimates for the model selection procedure. Simulations classified into the bottleneck model are shown in dark grey, whereas simulations classified into the non-bottleneck model are shown in light grey. A randomly chosen simulation generated under the bottleneck model was classified as a bottleneck model 87% of the time. A randomly chosen simulation that was generated under the non-bottleneck model was classified into the non-bottleneck model 94% of the time.

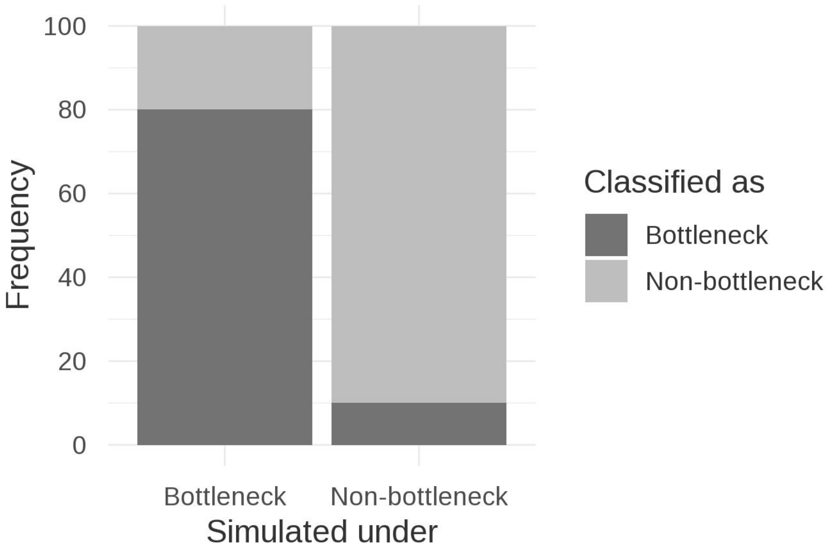

Fig S7. Posterior predictive checks for the summary statistics used in the ABC analysis. We sampled a set of 1,000 multivariate parameters from the posterior distributions that were estimated under the bottleneck model. Data were then re-simulated based on these parameters for each population under the bottleneck model to obtain 1,000 sets of summary statistics per species. Histograms show the distribution of the three summary statistics. The superimposed black lines represent the observed summary statistics calculated from the empirical data of each population (rarefied with 1,000 bootstraps).

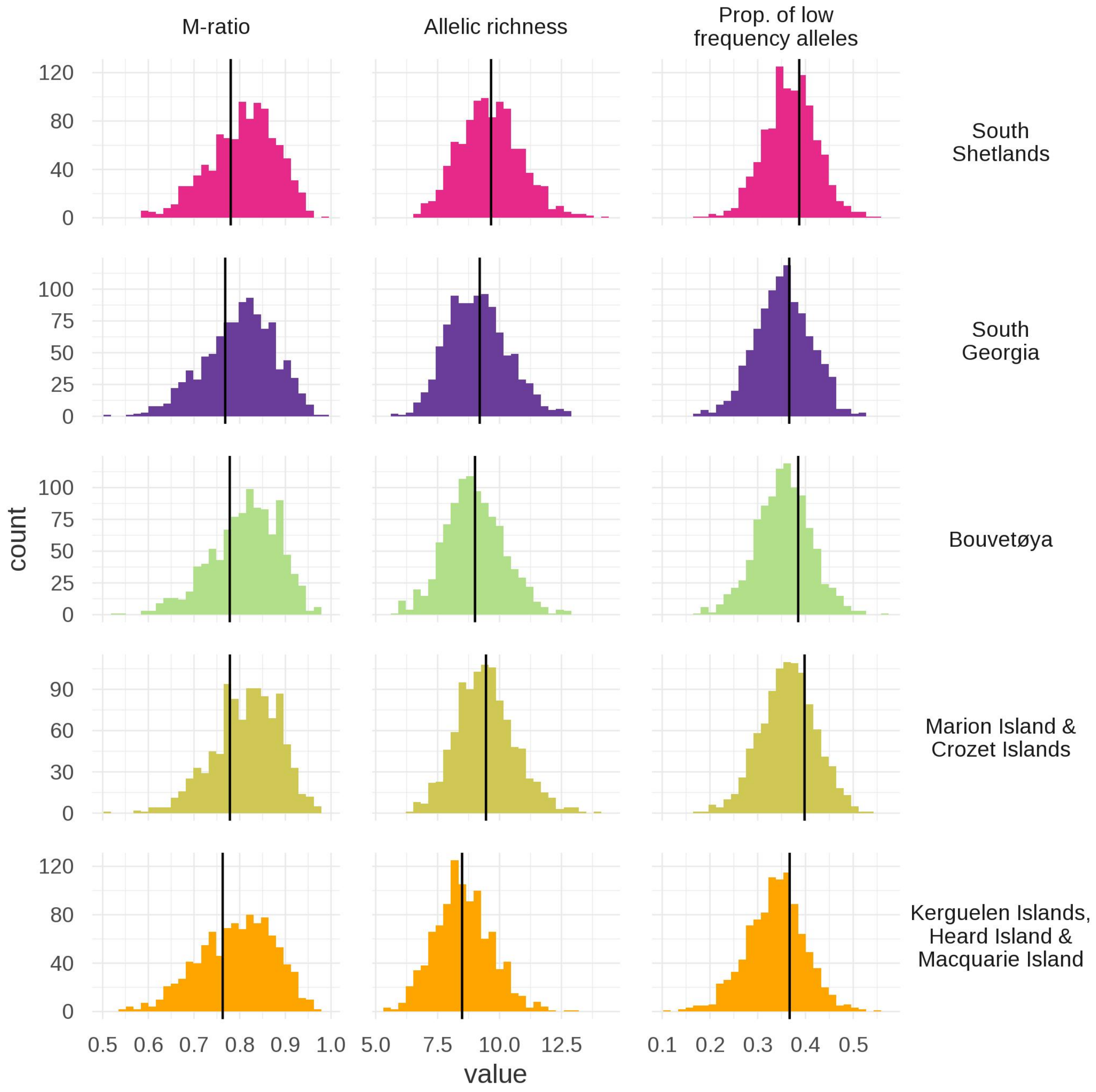

Fig. S8. Scatter plots of the of cross-validation evaluation of ABC model parameters (prediction errors are shown in **Table S4**). True values are plotted against the estimated values from our ABC analysis. The blue line indicates the expectation with a slope of one and an intercept of zero.

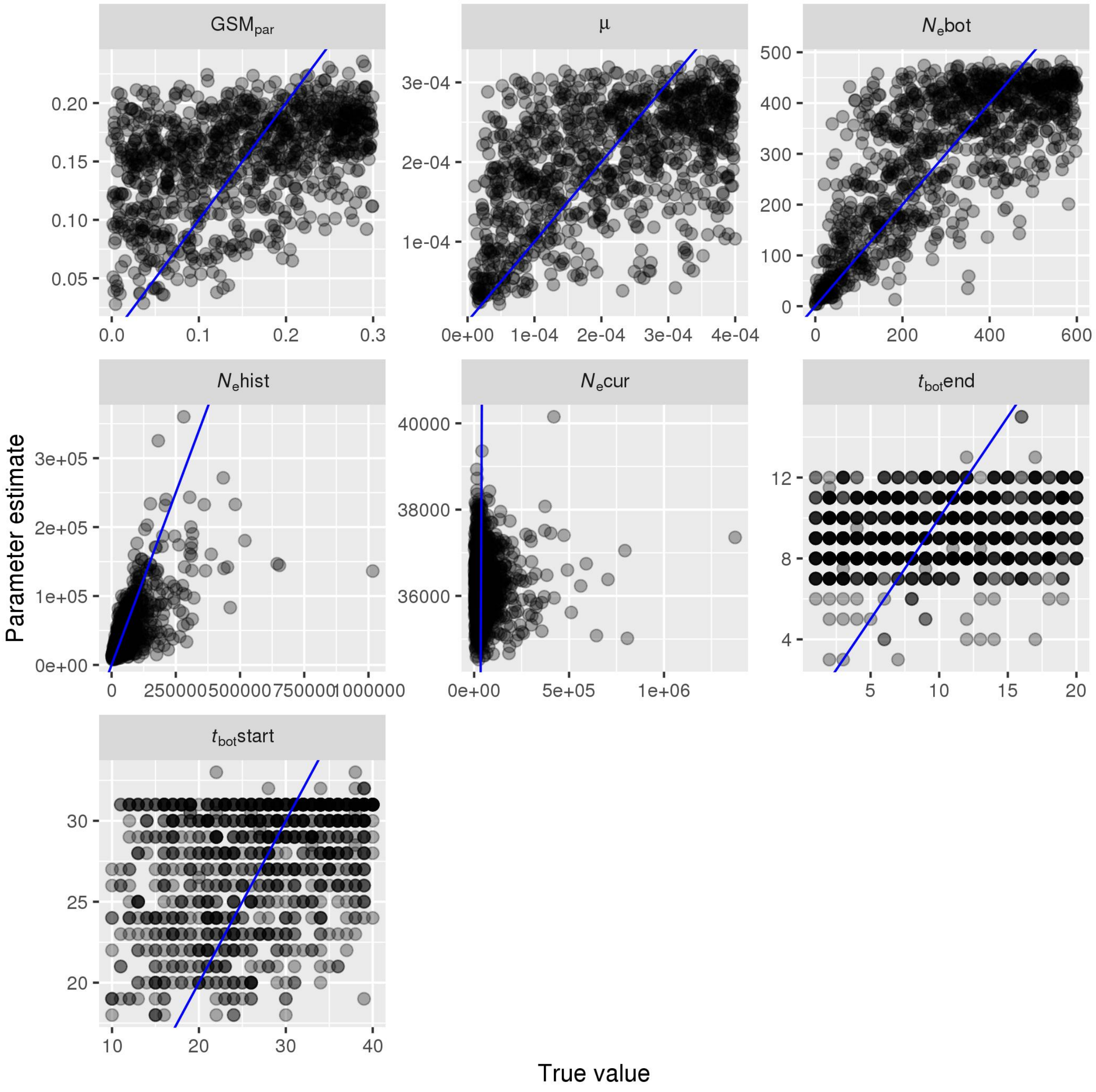

Fig. S9. Density plots of estimated historical effective population sizes ($N_e$hist). Shown are population-specific parameter values for 5,000 accepted simulations based on 18 individuals per population. Posterior values (colored lines) are superimposed over black dashed lines representing prior values. To facilitate visual inspection, the long tail of both posteriors and priors was cut off at $N_e$hist = 100,000. Note that although the prediction error for this parameter was relatively low, the cross-validation results show a systematic underestimation of the true values, especially when these are large. Consequently, these posteriors should be treated with caution.

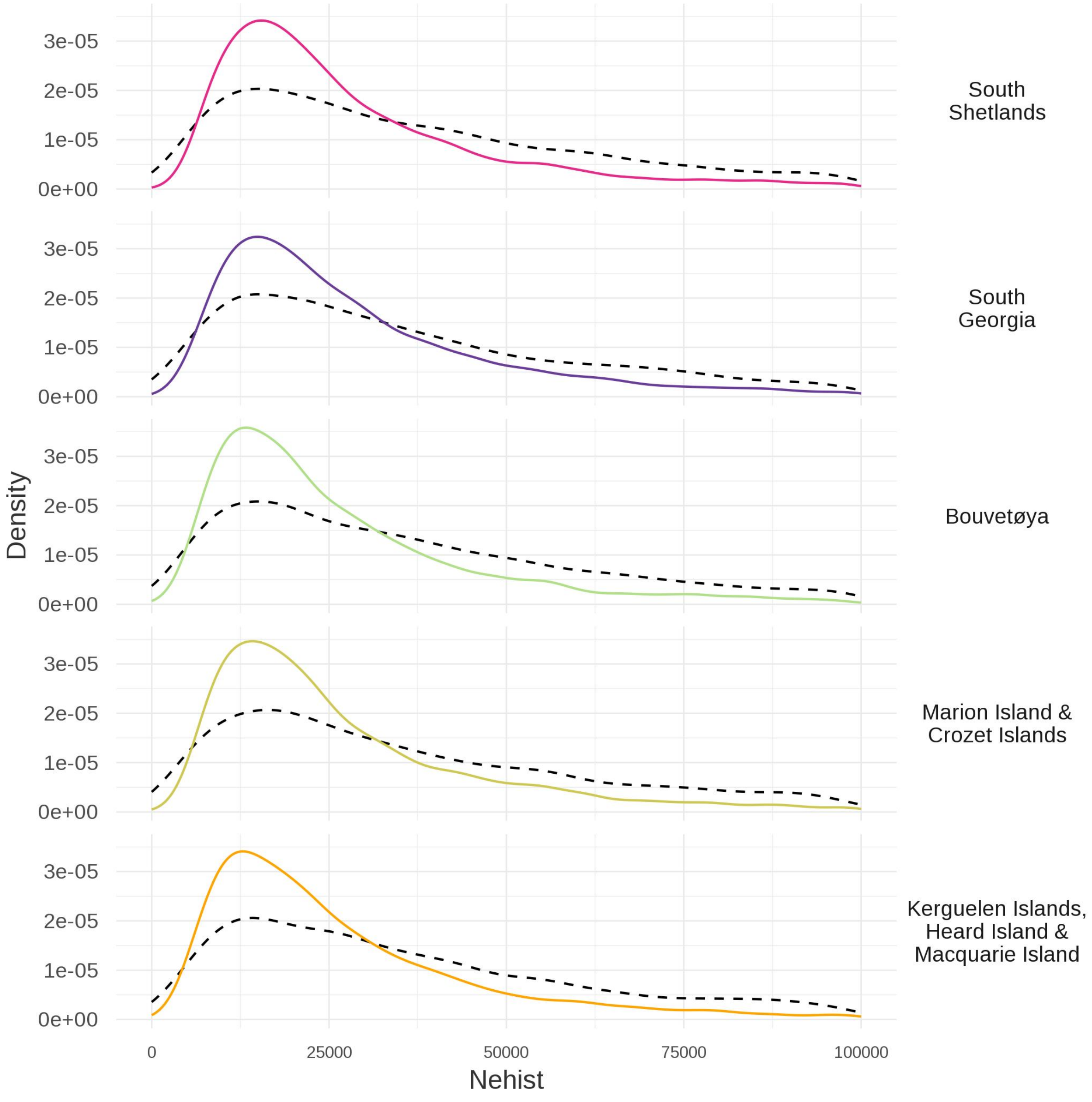

Fig. S10. Schematic representation of two contrasting demographic scenarios in our ABC analysis; (a) bottleneck scenario, (b) non-bottleneck scenario. All priors were drawn independently from each other, so the current N_e can be smaller or larger than $N_e$hist. This allowed both models to capture pre-bottleneck to post-bottleneck variation in population size. N_e and $N_e$hist were drawn from lognormal priors, whereas all of the other parameters were specified using uniform priors. t_{bot}start and t_{bot}end refer to the timing of the start and the end of the bottleneck respectively, while t_{hist} corresponds to the historical effective population size. All prior distributions are also shown as small figures next to the respective parameter. The exact priors and the mutation model are given in the Methods.

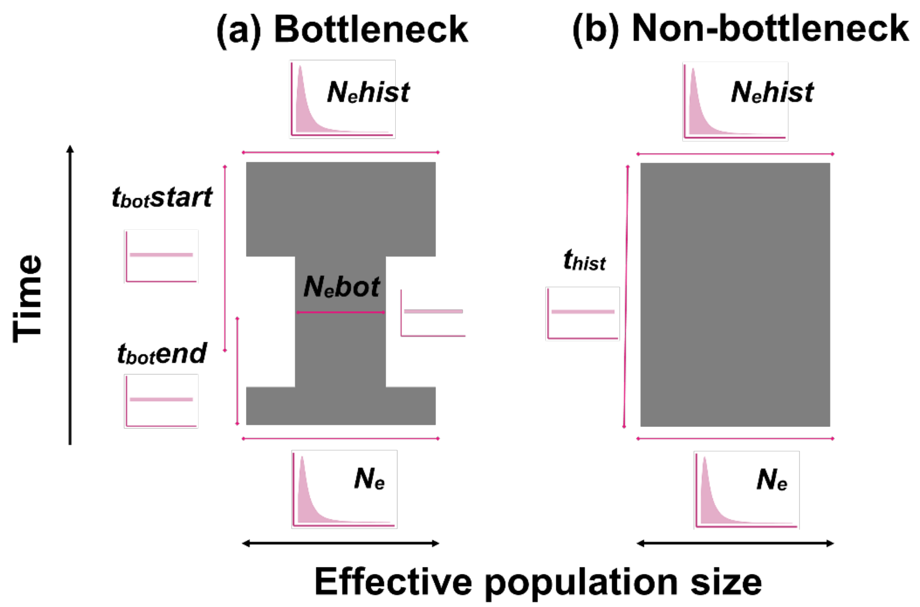

Table S1. Pairwise F_{st} values (below the diagonal) and associated 95% confidence intervals (above the diagonal, lower limit-upper limit) based on 1,000 bootstraps. F_{st} values not significantly different from zero are highlighted in bold.

	**South Shetlands**	**South Georgia**	**Bouvetøya**	**Marion Island**	**Crozet Islands**	**Kerguelen Islands**	**Heard Island**	**Macquarie Island**
**South Shetlands**		0.013 - 0.019	0.024 - 0.034	0.021 - 0.030	0.019 - 0.033	0.043 - 0.072	0.044 - 0.076	0.040 - 0.063
**South Georgia**	0.016		0.018 - 0.028	0.016 - 0.029	0.012 - 0.030	0.043 - 0.074	0.050 - 0.082	0.039 - 0.062
**Bouvetøya**	0.028	0.022		0.006 - 0.010	0.005 - 0.014	0.044 - 0.068	0.046 - 0.073	0.040 - 0.062
**Marion Island**	0.025	0.022	0.008		**-0.002 - 0.005**	0.017 - 0.030	0.018 - 0.034	0.017 - 0.029
**Crozet Islands**	0.025	0.021	0.009	**0.002**		0.018 - 0.038	0.019 - 0.039	0.018 - 0.037
**Kerguelen Islands**	0.057	0.059	0.057	0.024	0.027		**-0.002 - 0.005**	0.004 - 0.010
**Heard Island**	0.06	0.066	0.061	0.027	0.028	**0.001**		0.003 - 0.015
**Macquarie Island**	0.051	0.051	0.052	0.023	0.026	0.007	0.009	

Table S2. Genetic diversity and bottleneck signatures per sampling location. Rarefied allelic richness (A_r), observed heterozygosity (H_o), M-ratio and inbreeding coefficient F_{is} given as means and standard deviations (SD) across 39 loci. The numbers of private alleles are summed across loci. The number of loci with heterozygosity excess ($loci_{het\text{-}exc}$) and bottleneck test probabilities (Sign test, standardized differences tests and Wilcoxon test) are given under the two-phase model with 80% single-step mutations (TPM80) based on 1,000 iterations for each population.

**Population**	**GPS coordinates**	**Sample size**	A_r **(SD)**	H_o **(SD)**	**M-ratio (SD)**	F_{is} **(SD)**	**Private Alleles**	$\mathbf{Loci_{het\text{-}exc}}$	**Sign test *p*-value**	**Standardized differences test *p*-value**	**Wilcoxon test *p*-value**
South Shetlands	62 27S<br>60 47W	197	6.448<br>(2.555)	0.718<br>(0.197)	0.836<br>(0.185)	0.011<br>(0.046)	13	31	**0.005**	**<0.001**	**<0.001**
South Georgia	54 00S<br>38 03W	1,042	6.235<br>(2.506)	0.720<br>(0.207)	0.813<br>(0.200)	0.004<br>(0.026)	19	32	**0.001**	**<0.0001**	**<0.0001**
Bouvetøya	54 42S<br>3 41E	396	5.963<br>(2.470)	0.703<br>(0.223)	0.825<br>(0.195)	0.014<br>(0.047)	8	31	**0.004**	**<0.001**	**<0.0001**
Marion Island	46 54S<br>37 45E	166	6.295<br>(2.581)	0.719<br>(0.214)	0.830<br>(0.170)	0.005<br>(0.046)	2	31	**0.005**	**<0.001**	**<0.001**
Crozet Islands	46 40S<br>51 00E	18	5.985<br>(2.656)	0.718<br>(0.239)	0.822<br>(0.200)	-0.032<br>(0.122)	0	34	**<0.0001**	**<0.001**	**<0.0001**
Kerguelen Islands	49 36S<br>69 39E	51	5.407<br>(2.098)	0.688<br>(0.216)	0.827<br>(0.196)	-0.003<br>(0.090)	0	28	**0.046**	**0.004**	**0.002**
Heard Island	53 09S<br>73 58E	22	5.414<br>(2.146)	0.678<br>(0.218)	0.805<br>(0.203)	-0.021<br>(0.143)	1	25	0.276	0.115	0.117
Macquarie Island	54 64S<br>158 87E	108	5.570<br>(2.195)	0.689<br>(0.217)	0.823<br>(0.193)	0.001<br>(0.089)	5	27	0.099	**0.029**	**0.033**

Table S3. Model selection results for the ABC analysis showing posterior probabilities for the bottleneck model (p_{bot}) and neutral model (p_{neut}). Goodness of fit shows p-values for the respective models from 100 leave-one-out cross-validation replicates

Population	Posterior Probabilities		Goodness of fit	
	p_{bot}	p_{neut}	p_{bot}	p_{neut}
South Shetlands	0.990	0.010	0.900	0.200
South Georgia	0.998	0.002	0.990	0.140
Bouvetøya	0.985	0.015	0.910	0.160
Marion and Crozet Islands	0.980	0.020	0.920	0.170
Kerguelen, Heard and Macquarie Islands	0.995	0.005	1.000	0.100

Table S4. Prediction errors for current effective population size (N_e), bottleneck effective populations size ($N_e$bot), historical (pre-bottleneck) effective population size ($N_e$hist), start and end time for the bottleneck (t_{bot}start and t_{bot}start, respectively), mutation rate (μ) and the proportion of multistep mutations (GSMpar). The prediction error is based on a cross-validation sample of 1,000 under the bottleneck model. The smaller the value, the more information the posterior estimate contains about the true underlying parameter value.

N_e	$N_e$bot	$N_e$hist	t_{bot}end	t_{bot}start	μ	GSMpar
1.093	0.457	0.611	0.965	0.886	0.730	0.823

Table S5. Summary of ABC posterior estimates bottleneck for the effective populations size ($N_e$bot), historical (pre-bottleneck) effective population size ($N_e$hist) under the bottleneck model. The medians are based on 5.000 accepted parameter values for each species, while the modal values represent the respective modes of their density curves. Lower and upper CI represent the 95% bootstrapped confidence interval.

Population	$N_e$bot				$N_e$hist			
	Median	Mode	Lower _CI	Upper_ CI	Median	Mode	Lower _CI	Upper_ CI
South Shetlands	237	191	34	592	23,029	17,274	5,697	208,468
South Georgia	203	161	29	588	23,863	17,619	5,093	184,777
Bouvetøya	226	170	30	591	21,255	16,477	4,688	180,333
Marion and Crozet Islands	243	187	33	591	22,094	17,073	5,199	204,316
Kerguelen, Heard and Macquarie Islands	189	144	23	589	21,751	16,107	4,455	174,648

SI methods and results–microsatellite genotyping and hybrid analyses

SI Methods

Microsatellite genotyping

PCR amplification of the microsatellite loci was performed in five separate multiplexed reactions using a Type It Kit (Qiagen) and the following PCR profile: one cycle of 5 min at 94°C; 24 cycles of 30 s at 94°C, 90 s at T_a°C, 30 s at 72°C; and one final cycle of 15 min at 72°C (see **Table S6** for mastermix-specific annealing temperatures, T_a). Fluorescently labelled PCR products were resolved by electrophoresis on an ABI 3730xl capillary sequencer (Applied Biosystems, Waltham, MA, USA) and allele sizes were scored automatically using GeneMarker v. 2.6.2 (SoftGenetics, LLC., State College, PA, USA). All traces were manually inspected and corrected where necessary to ensure high genotype data quality. Four positive controls were included on each plate to facilitate the standardisation of microsatellite scoring across plates, and occasional negative controls were also included, none of which yielded interpretable PCR products.

Table S6. PCR conditions used to amplify 39 microsatellites. "Multiplex" denotes the PCR mastermix into which each locus was multiplexed and "T_a" denotes the annealing temperature used.

Locus	Reference	Multiplex	T_a in °C
Pv9	Allen et al. (1995)	1	53°C
Hg6.3	Allen et al. (1995)	1	53°C
Hg8.10	Allen et al. (1995)	1	53°C
Hg1.3	Gemmell et al. (1997)	1	53°C
M11a	Hoelzel et al. (1999)	1	53°C
PvcA	Coltman et al. (1996)	1	53°C
Zcwb07	Hoffman et al. (2007)	1	53°C
Agaz2	Hoffman (2009)	1	53°C
Ag3	Hoffman et al. (2008)	2	60°C
Agaz6	Hoffman (2009)	2	60°C
OrrFCB7	Buchanan et al. (1998)	2	60°C
Ag2	Hoffman et al. (2008)	2	60°C
OrrFCB2	Buchanan et al. (1998)	2	60°C
Lw10	Davis et al. (2002)	2	60°C
ZcwC01	Hoffman et al. (2007)	2	60°C
Agaz5	Hoffman (2009)	2	60°C
Zcwb.14	Hernandez-Velazquez et al. (2005)	2	60°C
Ssl301	Huebinger et al. (2007)	3	60°C
Ag7	Hoffman et al. (2008)	3	60°C
Agt10	Hoffman et al. (2008)	3	60°C

(table continues on the next page)

Locus	Reference	Multiplex	T_a in °C
ZcwCgDh4.7	Hernandez-Velazquez et al. (2005)	3	60°C
ZcwE05	unpublished	3	60°C
Ag1	Hoffman et al. (2008)	3	60°C
OrrFCB8	Buchanan et al. (1998)	3	60°C
Agt-47	Hoffman and Nichols (2011)	3	60°C
ZcwF07	Hoffman et al. (2007)	4	53°C
ZcwD02	Wolf et al. (2006)	4	53°C
ZcwCgDh1.8	Hernandez-Velazquez et al. (2005)	4	53°C
Aa4	Hoelzel et al. (1999)	4	53°C
ZcwCgDh5.8	Hernandez-Velazquez et al. (2005)	4	53°C
Agaz3	Hoffman (2009)	4	53°C
962-1	Stoffel et al. (2018)	5	60°C
554-6	Stoffel et al. (2018)	5	60°C
ZcwA12F	Hoffman et al. (2007)	5	60°C
PvcE	Coltman et al. (1996)	5	60°C
ZcwB09	Wolf et al. (2006)	5	60°C
Agaz10	Hoffman (2009)	5	60°C
Mang44	Sanvito et al. (2013)	5	60°C
Mang36	Sanvito et al. (2013)	5	60°C

Identification and removal of putative hybrids

Antarctic fur seals are known to hybridise with the Subantarctic fur seal (*A. tropicalis*) on at least three of sub-Antarctic islands: Marion Island [1], Crozet Islands [2] and Macquarie Island[3]. In addition, vagrant Subantarctic fur seals have been observed on Bouvetøya [4] and Heard Island [5]. As the inclusion of inadvertently sampled *A. tropicalis* individuals or hybrids in our dataset could potentially affect downstream analyses, STRUCTURE version 2.3.3 [6] was used to identify individuals with a high proportion of *A. tropicalis* ancestry. STRUCTURE has been shown to be more robust than NewHybrids when the frequency of hybridization is low [7]. As clustering analyses such as STRUCTURE can be sensitive to missing data, all animals with missing genotypes for more than 4 of 39 loci were removed. This resulted in a dataset of 2,155 individuals comprising 2,064 animals that were identified by field researchers as Antarctic fur seals plus 91 animals from a reference population of *A. tropicalis.* STRUCTURE assumes all loci to be in Hardy Weinberg Equilibrium (HWE) and linkage equilibrium. Thus, deviations from HWE and linkage disequilibrium (LD) were tested for, separately for each species and population. The Monte Carlo procedure in the package adegenet [8,9] in R was used to test for deviations from HWE with 10,000 replicates. LD was tested using Genepop (Rousset 2008) with 1,000 dememorizations, 100 batches, and 1,000 iterations per batch. As the p-values were not uniformly distributed for all populations, the Bonferroni correction in the R-package qvalue [10] was used to adjust significance values accordingly. Four loci were out of HWE in the Subantarctic fur seal (OrrFCB7, Lw10,

SSL301 and ZcwD02) and were therefore removed before testing for hybrids. Additionally, a fifth locus (Mang44) was also removed because it did not amplify well in the Subantartic fur seals.

STRUCTURE was run using the R-package ParallelStructure [11] with the following parameters: 500,000 burn-in length, 1,000,000 MCMC replications, 1–3 assumed populations (*k*) and ten iterations for each *k*. The other parameters were kept at default values. A two population model (i.e. $k = 2$) was used to classified individuals that were admixed with at least 10% of the genetic attribution being to the secondary species (i.e. $0.10 \le q \le 0.9$) [see also 7]. In addition to STRUCTURE, a confirmatory analysis was also conducted using principal component analysis (PCA) in the adegenet package. Because PCA is sensitive to missing data, missing genotypes were imputed [12] and allele frequencies were transformed by centring the data.

SI Results

Hybrid analyses

Bayesian analysis of the full dataset within STRUCTURE clearly separated *A. gazella* from *A. tropicalis* (**Fig. S11**). In addition, 49 individuals that were sampled as *A. gazella* were classified by the cluster analysis ($q_{AFS} < 0.1$) as pure *A. tropicalis*, whereas five individuals sampled as *A. tropicalis* were classified as hybrids. A further 15 Antarctic fur seal individuals had a q value between 0.10 and 0.90 and were therefore classified as hybrids. Similarly, PCA clearly separated *A. gazella* from *A. tropicalis*, while the putative hybrids fell in between (**Fig. S12**).

For the final dataset of 2,000 pure Antarctic fur seal individuals, none of the microsatellite loci were out of HWE in more than two of the eight populations (**Table S7**), and no pairs of loci were in LD in more than three of the eight populations. Consequently, all 39 loci were retained for subsequent data analyses.

Fig. S11. Results of the hybrid analysis of the full dataset of 2,155 fur seals within STRUCTURE (see SI methods for details). Each individual is represented by a vertical bar with the proportion of the colouring representing the probability of belonging to each cluster (dark grey = *A. gazella*, light grey = *A. tropicalis*). Individuals are grouped by sampling location, from left to right: South Shetland Islands (SSI), South Georgia (SG), Bouvetøya (BI), Marion Island (MAR), Crozet Islands (CI), Kerguelen Islands (KI), Heard Island (HI), Macquarie Island (MAC) and *A. tropicalis* from Macquarie Island (TM). Individuals with at least 10% admixture were classified as putative hybrids and are indicated by black arrows.

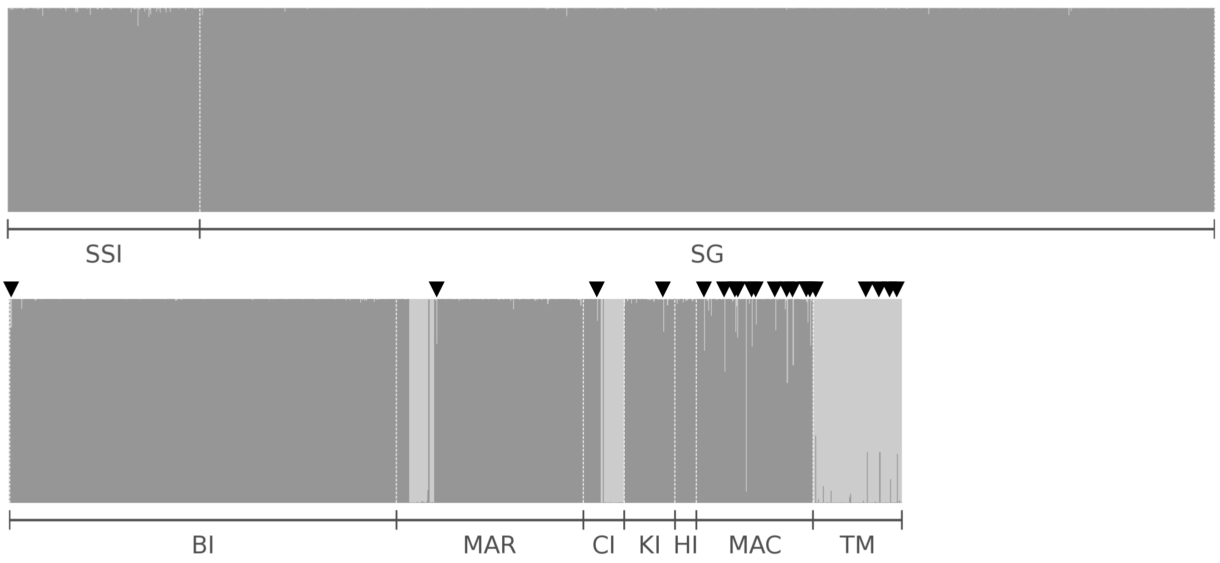

Fig. S12. Genetic clustering of species visualised by PCA of 34 microsatellites. Points represent individual variation in (a) principal components one and two; and (b) principal components one and three. Colours represent species assignments according to STRUCTURE as shown in the legend.

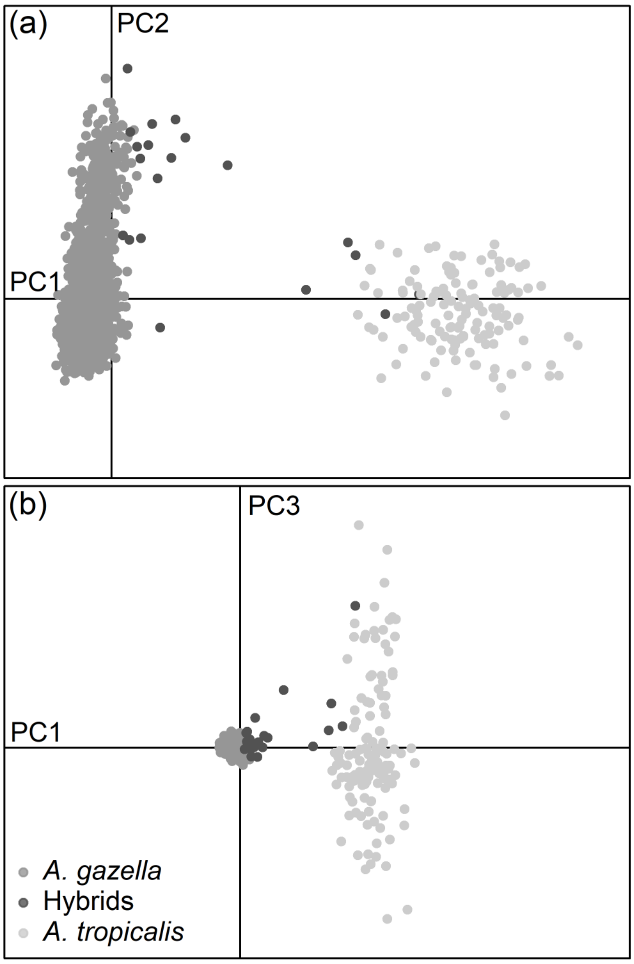

Table S7. Observed heterozygosity (H_o), expected heterozygosity (H_e), uncorrected exact test results based on 10,000 Monte Carlo permutations (*p*-exact) and Bonferroni corrected *p*-values. *P*-values that remained significant after correction for multiple tests are highlighted in bold and underlined.

	**South Shetlands (*n* = 197)**				**South Georgia (*n* = 1,042)**				**Bouvetoya (*n* = 396)**				**Marion Island (*n* = 166)**			
**Locus**	***Ho***	***He***	***p*-exact**	***p*-value**	***Ho***	***He***	***p*-exact**	***p*-value**	***Ho***	***He***	***p*-exact**	***p*-value**	***Ho***	***He***	***p*-exact**	***p*-value**
Pv9	0.756	0.778	0.193	1.000	0.768	0.766	0.083	1.000	0.810	0.827	0.928	1.000	0.837	0.841	0.521	1.000
Hg63	0.918	0.896	0.414	1.000	0.861	0.869	0.224	1.000	0.848	0.841	0.205	1.000	0.878	0.853	0.525	1.000
Hg810	0.396	0.407	0.170	1.000	0.402	0.444	0.056	1.000	0.492	0.500	0.264	1.000	0.506	0.514	0.625	1.000
Hg13	0.897	0.866	0.348	1.000	0.853	0.863	0.662	1.000	0.848	0.838	0.232	1.000	0.843	0.840	0.778	1.000
M11a	0.856	0.890	0.280	1.000	0.915	0.915	0.987	1.000	0.859	0.909	0.122	1.000	0.823	0.913	0.002	0.078
PvcA	0.755	0.778	0.207	1.000	0.786	0.790	0.003	0.125	0.799	0.817	0.487	1.000	0.867	0.826	0.351	1.000
Zcwb07	0.787	0.829	0.132	1.000	0.864	0.864	0.819	1.000	0.796	0.780	0.962	1.000	0.805	0.819	0.660	1.000
Agaz2	0.813	0.817	0.053	1.000	0.823	0.802	0.507	1.000	0.823	0.823	0.642	1.000	0.898	0.843	0.766	1.000
Ag3	0.406	0.358	0.074	1.000	0.298	0.285	0.158	1.000	0.187	0.194	0.442	1.000	0.145	0.154	0.321	1.000
Agaz6	0.599	0.591	0.232	1.000	0.619	0.623	0.976	1.000	0.509	0.518	0.888	1.000	0.473	0.521	0.170	1.000
OrrFCB7	0.831	0.860	0.186	1.000	0.861	0.849	0.478	1.000	0.774	0.778	0.994	1.000	0.812	0.785	0.179	1.000
Ag2	0.724	0.779	0.196	1.000	0.768	0.780	0.720	1.000	0.628	0.658	0.008	0.300	0.733	0.720	0.278	1.000
OrrFCB2	0.805	0.883	0.001	**0.035**	0.841	0.859	0.761	1.000	0.863	0.856	0.865	1.000	0.898	0.885	0.559	1.000
Lw10	0.892	0.908	0.377	1.000	0.897	0.904	0.039	1.000	0.929	0.901	0.689	1.000	0.921	0.902	0.040	1.000
Zcwc0103	0.832	0.863	0.295	1.000	0.853	0.861	0.162	1.000	0.828	0.845	0.007	0.254	0.829	0.866	0.018	0.694
Agaz5	0.490	0.583	0.001	**0.020**	0.496	0.530	0.000	**0.000**	0.631	0.652	0.354	1.000	0.675	0.684	0.684	1.000
ZcwCgDhB14	0.711	0.756	0.058	1.000	0.764	0.778	0.533	1.000	0.775	0.785	0.254	1.000	0.765	0.790	0.842	1.000
SSL301	0.873	0.883	0.674	1.000	0.902	0.888	0.026	0.998	0.827	0.830	0.694	1.000	0.861	0.846	0.340	1.000
Ag7	0.753	0.771	0.797	1.000	0.752	0.753	0.424	1.000	0.670	0.692	0.026	1.000	0.742	0.750	0.875	1.000
Agt10	0.269	0.266	0.593	1.000	0.327	0.348	0.059	1.000	0.181	0.248	0.000	**0.000**	0.299	0.304	0.573	1.000
ZcwCgDh47	0.832	0.862	0.583	1.000	0.879	0.879	0.824	1.000	0.885	0.869	0.498	1.000	0.902	0.881	0.224	1.000
Zcwe05	0.822	0.821	0.032	1.000	0.776	0.780	0.454	1.000	0.815	0.798	0.800	1.000	0.742	0.725	0.192	1.000
Ag1	0.883	0.876	0.353	1.000	0.884	0.871	0.344	1.000	0.835	0.848	0.173	1.000	0.800	0.807	0.105	1.000
OrrFCB8	0.781	0.809	0.203	1.000	0.779	0.783	0.902	1.000	0.769	0.773	0.017	0.675	0.799	0.774	0.692	1.000
Agt47	0.396	0.383	0.109	1.000	0.499	0.520	0.218	1.000	0.562	0.577	0.146	1.000	0.588	0.558	0.383	1.000
Zcwf07	0.858	0.829	0.132	1.000	0.766	0.765	0.735	1.000	0.843	0.831	0.003	0.098	0.812	0.836	0.554	1.000
ZcwD02	0.863	0.859	0.582	1.000	0.851	0.854	0.880	1.000	0.878	0.866	0.037	1.000	0.910	0.864	0.141	1.000
ZcwCgDh18	0.772	0.751	0.250	1.000	0.754	0.753	0.557	1.000	0.715	0.710	0.612	1.000	0.801	0.759	0.838	1.000
Aa4	0.787	0.757	0.986	1.000	0.726	0.739	0.631	1.000	0.725	0.711	0.303	1.000	0.663	0.707	0.637	1.000
ZcCgDh58	0.868	0.867	0.234	1.000	0.872	0.872	0.569	1.000	0.812	0.821	0.943	1.000	0.855	0.865	0.407	1.000
Agaz3	0.675	0.674	0.536	1.000	0.612	0.628	0.797	1.000	0.669	0.686	0.214	1.000	0.705	0.672	0.451	1.000
X9621MHCI	0.597	0.601	0.706	1.000	0.561	0.569	0.000	**0.000**	0.529	0.541	0.280	1.000	0.540	0.558	0.940	1.000
X5546MHCI	0.223	0.233	0.019	0.725	0.141	0.139	0.825	1.000	0.068	0.066	1.000	1.000	0.181	0.185	0.006	0.218
Zcwa12	0.853	0.884	0.108	1.000	0.807	0.844	0.015	0.581	0.879	0.889	0.340	1.000	0.849	0.897	0.381	1.000
PvcE	0.908	0.878	0.063	1.000	0.868	0.879	0.017	0.667	0.843	0.866	0.198	1.000	0.855	0.887	0.278	1.000
Zcwb09	0.838	0.813	0.316	1.000	0.860	0.859	0.129	1.000	0.884	0.879	0.795	1.000	0.885	0.884	0.536	1.000
agaz10	0.796	0.799	0.202	1.000	0.777	0.781	0.988	1.000	0.797	0.812	0.356	1.000	0.819	0.819	0.088	1.000
Mang44	0.682	0.700	0.549	1.000	0.735	0.732	0.677	1.000	0.712	0.711	0.969	1.000	0.606	0.699	0.050	1.000
Mang36	0.225	0.225	0.540	1.000	0.138	0.142	0.037	1.000	0.105	0.105	0.714	1.000	0.133	0.126	1.000	1.000

(table continues on the next page)

	Crozet Islands (n = 18)				Kerguelen Islands (n = 51)				Heard Island (n = 22)				Macquarie Island (n = 108)			
**Locus**	***Ho***	***He***	***p*-exact**	***p*-value**	***Ho***	***He***	***p*-exact**	***p*-value**	***Ho***	***He***	***p*-exact**	***p*-value**	***Ho***	***He***	***p*-exact**	***p*-value**
Pv9	0.722	0.824	0.641	1.000	0.863	0.821	0.003	0.113	1.000	0.826	0.299	1.000	0.824	0.816	0.991	1.000
Hg63	0.944	0.863	0.839	1.000	0.863	0.812	0.182	1.000	0.864	0.842	0.553	1.000	0.852	0.861	0.272	1.000
Hg810	0.333	0.401	0.568	1.000	0.549	0.484	0.399	1.000	0.364	0.500	0.211	1.000	0.509	0.490	0.844	1.000
Hg13	0.889	0.835	0.493	1.000	0.880	0.809	0.848	1.000	0.727	0.751	0.189	1.000	0.824	0.806	0.203	1.000
M11a	0.778	0.887	0.499	1.000	0.667	0.814	0.004	0.144	0.773	0.759	0.205	1.000	0.786	0.763	0.678	1.000
PvcA	0.889	0.733	0.891	1.000	0.765	0.774	0.974	1.000	0.864	0.793	0.823	1.000	0.796	0.745	0.320	1.000
Zcwb07	0.944	0.731	0.880	1.000	0.843	0.789	0.676	1.000	0.905	0.773	0.468	1.000	0.769	0.754	0.988	1.000
Agaz2	0.944	0.833	0.827	1.000	0.796	0.851	0.625	1.000	0.818	0.838	0.351	1.000	0.870	0.850	0.859	1.000
Ag3	0.333	0.278	1.000	1.000	0.098	0.093	1.000	1.000	0.091	0.087	1.000	1.000	0.065	0.080	0.161	1.000
Agaz6	0.556	0.681	0.515	1.000	0.255	0.348	0.010	0.390	0.238	0.425	0.002	0.070	0.269	0.469	0.000	**0.000**
OrrFCB7	0.833	0.821	0.570	1.000	0.922	0.837	0.611	1.000	0.864	0.825	0.937	1.000	0.804	0.814	0.689	1.000
Ag2	0.722	0.733	0.647	1.000	0.740	0.767	0.331	1.000	0.818	0.735	0.973	1.000	0.824	0.788	0.469	1.000
OrrFCB2	0.824	0.862	0.261	1.000	0.822	0.776	0.711	1.000	0.667	0.717	0.308	1.000	0.785	0.838	0.354	1.000
Lw10	0.944	0.878	0.672	1.000	0.900	0.861	0.831	1.000	0.773	0.815	0.210	1.000	0.907	0.902	0.505	1.000
Zcwc0103	0.727	0.822	0.719	1.000	0.844	0.767	0.848	1.000	0.813	0.750	0.601	1.000	0.786	0.804	0.671	1.000
Agaz5	0.611	0.674	0.484	1.000	0.660	0.619	0.699	1.000	0.500	0.526	0.273	1.000	0.626	0.695	0.165	1.000
ZcwCgDhB14	0.722	0.715	0.496	1.000	0.800	0.749	0.187	1.000	0.818	0.714	0.416	1.000	0.796	0.752	0.916	1.000
SSL301	0.778	0.835	0.355	1.000	0.824	0.806	0.682	1.000	0.773	0.717	0.984	1.000	0.778	0.762	0.929	1.000
Ag7	0.941	0.785	0.495	1.000	0.882	0.844	0.454	1.000	0.727	0.837	0.125	1.000	0.806	0.822	0.588	1.000
Agt10	0.111	0.106	1.000	1.000	0.451	0.480	0.404	1.000	0.318	0.280	1.000	1.000	0.370	0.351	0.470	1.000
ZcwCgDh47	0.944	0.860	0.905	1.000	0.882	0.840	0.482	1.000	0.773	0.792	0.774	1.000	0.850	0.861	0.827	1.000
Zcwe05	0.833	0.708	0.428	1.000	0.373	0.412	0.269	1.000	0.545	0.507	0.836	1.000	0.565	0.537	0.105	1.000
Ag1	0.833	0.779	0.988	1.000	0.804	0.748	0.847	1.000	0.773	0.732	0.795	1.000	0.776	0.794	0.062	1.000
OrrFCB8	0.722	0.767	0.077	1.000	0.706	0.731	0.662	1.000	0.818	0.725	0.331	1.000	0.657	0.652	0.649	1.000
Agt47	0.500	0.563	0.330	1.000	0.627	0.584	0.611	1.000	0.682	0.520	0.442	1.000	0.565	0.559	0.988	1.000
Zcwf07	0.889	0.809	0.966	1.000	0.706	0.715	0.964	1.000	0.682	0.746	0.043	1.000	0.766	0.762	0.997	1.000
ZcwD02	0.706	0.782	0.106	1.000	0.824	0.830	0.508	1.000	0.727	0.838	0.055	1.000	0.796	0.819	0.852	1.000
ZcwCgDh18	0.667	0.728	0.577	1.000	0.686	0.644	0.009	0.351	0.773	0.727	0.976	1.000	0.722	0.696	0.798	1.000
Aa4	0.722	0.637	0.810	1.000	0.706	0.710	0.028	1.000	0.636	0.683	0.766	1.000	0.720	0.676	0.916	1.000
ZcCgDh58	0.889	0.869	0.949	1.000	0.882	0.864	0.908	1.000	0.818	0.855	0.702	1.000	0.880	0.873	0.093	1.000
Agaz3	0.778	0.620	0.543	1.000	0.694	0.679	0.738	1.000	0.864	0.688	0.256	1.000	0.769	0.687	0.117	1.000
X9621MHCI	0.389	0.424	1.000	1.000	0.457	0.498	0.573	1.000	0.545	0.491	0.343	1.000	0.565	0.509	0.001	**0.027**
X5546MHCI	0.222	0.198	1.000	1.000	0.196	0.237	0.215	1.000	0.227	0.201	1.000	1.000	0.185	0.183	1.000	1.000
Zcwa12	0.889	0.903	0.260	1.000	0.706	0.870	0.005	0.211	0.682	0.848	0.088	1.000	0.815	0.856	0.165	1.000
PvcE	0.944	0.875	0.975	1.000	0.824	0.859	0.496	1.000	0.810	0.876	0.307	1.000	0.877	0.867	0.126	1.000
Zcwb09	0.944	0.866	0.818	1.000	0.686	0.721	0.347	1.000	0.773	0.754	0.807	1.000	0.833	0.782	0.938	1.000
agaz10	0.722	0.761	0.140	1.000	0.804	0.760	0.354	1.000	0.818	0.698	0.547	1.000	0.787	0.760	0.779	1.000
Mang44	0.813	0.703	0.419	1.000	0.682	0.666	0.907	1.000	0.600	0.648	0.008	0.296	0.636	0.657	0.883	1.000
Mang36	0.063	0.061	1.000	1.000	0.163	0.151	1.000	1.000	0.182	0.165	1.000	1.000	0.093	0.089	1.000	1.000

References

1. Hofmeyr, G. J. G., Bester, M. N., Makhado, A. B. & Pistorius, P. A. Population changes in Subantarctic and Antarctic fur seals at Marion Island. *South Afr. J. Wildl. Res.* **36**, 55–68 (2006).
2. Kingston, J. J. & Gwilliam, J. Hybridization between two sympatrically breeding species of fur seal at Iles Crozet revealed by genetic analysis. *Conserv. Genet.* **8**, 1133 (2007).
3. Lancaster, M. L., Gemmell, N. J., Negro, S., Goldsworthy, S. & Sunnucks, P. Ménage à trois on Macquarie Island: hybridization among three species of fur seal (Arctocephalus spp.) following historical population extinction. *Mol. Ecol.* **15**, 3681–3692 (2006).
4. Hofmeyr, G. J. G., Bester, M. N. & Kirkman, S. P. Vagrant Subantarctic fur seals at Bouvetøya. *Afr. Zool.* **41**, 145–146 (2006).
5. Page, B. *et al.* Population status and breeding season chronology of Heard Island fur seals. *Polar Biol.* **26**, 219–224 (2003).
6. Pritchard, J. K., Stephens, M. & Donnelly, P. Inference of population structure using multilocus genotype data. *Genetics* **155**, 945–959 (2000).
7. Vähä, J.-P. & Primmer, C. R. Efficiency of model-based Bayesian methods for detecting hybrid individuals under different hybridization scenarios and with different numbers of loci. *Mol. Ecol.* **15**, 63–72 (2005).
8. Jombart, T. adegenet: a R package for the multivariate analysis of genetic markers. *Bioinformatics* **24**, 1403–1405 (2008).
9. Jombart, T. & Ahmed, I. adegenet 1.3-1: new tools for the analysis of genome-wide SNP data. *Bioinformatics* **27**, 3070–3071 (2011).
10. Storey, J. D., Bass, A. J., Dabney, A. & Robinson, D. *qvalue: Q-value estimation for false discovery rate control.* (2015).
11. Besnier, F. & Glover, K. A. ParallelStructure: a R package to distribute parallel runs of the population genetics program STRUCTURE on multi-core computers. *PLOS ONE* **8**, e70651 (2013).
12. Jombart, T., Devillard, S. & Balloux, F. Discriminant analysis of principal components: a new method for the analysis of genetically structured populations. *BMC Genet.* **11**, 94 (2010).
